# Luminescence and energy transfer processes in Gd_0.99_Er_0.01_Al_0.995_Cr_0.05_O_3_

**DOI:** 10.1039/d5ra04226g

**Published:** 2025-08-19

**Authors:** F. Mselmi, Abir Hadded, Hajer Souissi, Souha Kammoun, J. Pina, B. F. O. Costa

**Affiliations:** a Laboratoire de Physique Appliquée, Faculté des Sciences, Université de Sfax 3000 Tunisia abir.hadded1994@gmail.com; b University of Coimbra, CQC-IMS, Chemistry Department, Rua Larga P-3004-535 Coimbra Portugal; c University of Coimbra, CFisUC, Physics Department, Rua Larga P-3004-516 Coimbra Portugal

## Abstract

Gd_0.99_Er_0.01_AlO_3_ and Gd_0.99_Er_0.01_Al_0.995_Cr_0.05_O_3_ samples were synthesized using a solid-state reaction method. Structural analysis revealed that the samples crystallized in an orthorhombic structure phase with a *Pbnm* space group. The average crystallite sizes were around 283 nm and 574 nm for Gd_0.99_Er_0.01_AlO_3_ and Gd_0.99_Er_0.01_Al_0.995_Cr_0.05_O_3_, respectively. Derivative absorption spectrum fitting (DASF) and first-derivative reflectance (d*R* /d*λ*) methods confirmed that the samples possess a direct wide band gap, with energies of 5.93 eV and 5.90 eV, respectively. The photoluminescence (PL) spectrum of Gd_0.99_Er_0.01_AlO_3_ under *λ*_ex_ = 377 nm excitation exhibits a green emission and intense sharp red lines at 680 nm, 697 nm, 705 nm, 717 nm and 758 nm. The green emission corresponds to the transitions ^2^H_11/2_ → ^4^I_15/2_ and ^4^S_3/2_ → ^4^I_15/2_ of Er^3+^ ions, while the sharp red lines are attributed to transitions between intrinsic defect centers related to the GdAlO_3_ host coupled to B_3g_ (4) and B_1g_ (7) vibrational modes. Efficient energy transfer *via* resonant phonon-assisted and cross-relaxation processes from Er^3+^ and intrinsic defect centers to Cr^3+^ is responsible for the decrease in green and red emission line intensities in Gd_0.99_Er_0.01_Al_0.995_Cr_0.05_O_3_. The energy transfer from Er^3+^ and intrinsic defect centers indicates that red emission lines at 697 nm and 726 nm in Gd_0.99_Er_0.01_Al_0.995_Cr_0.05_O_3_ mainly originate from the ^2^T_1_ (^2^G) → ^4^A_2_ (^4^F) and ^2^E_g_ (^2^G) → ^4^A_2g_ (^4^F) transitions of Cr^3+^ ions.

## Abstract 2

Gd_0.99_Er_0.01_AlO_3_ and Gd_0.99_Er_0.01_Al_0.995_Cr_0.05_O_3_ samples were synthesized using a solid-state reaction method.

## Graphical abstract

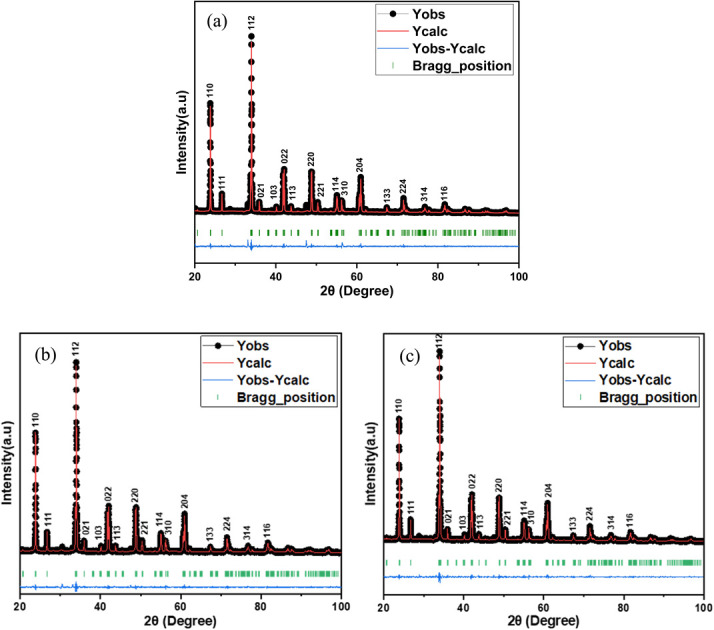

## 1. Introduction

Perovskite compounds serve as excellent host materials for various optical applications due to their chemical and thermal stability.^1,2^ They follow the generic formula ABO_3_. Rare-earth orthoaluminates (REAlO_3_), such as gadolinium aluminate (GdAlO_3_), possess significant optical, thermal, and mechanical properties, making them appropriate as solid-state laser hosts.^3^ GdAlO_3_ is also known for its relatively high dielectric constant, making it valuable for electronic applications, and is being developed as a potential material for neutron absorption and control rod applications.^4^ In cubic perovskites, the tolerance factor is *t*_obs=1_,^5^ whereas for GdAlO_3_ with the *Pbnm* space group, *t*_obs_ = 0.986,^6^ indicating a slight distortion from the cubic structure. GdAlO_3_, formed with Gd^3+^ ions having a relatively large ion radius (180.4 pm), closely approaches the ideal cubic perovskite crystal cell (*Pm*3̄*m*). GdAlO_3_ with the *Pbnm* space group demonstrates a high accommodation capacity within the perovskite structure and assists in the modulation of its electronic and spectroscopic properties through the substitution of Gd^3+^ with various rare-earth activators (*e.g.*, Eu^3+^, Er^3+^, Tb^3+^, Ce^3+^, Yb^3+^, Dy^3+^) and Al^3+^ with a transition metal activator ion such as Cr^3+^ and Mn^4+^. In this structure, the Gd^3+^ ion occupies a non-centrosymmetric site, leading to mixing of the 4f^*n*^ states with the first excited configuration 4f^*n*−1^5d. This mixing is caused by the odd terms in the crystal field, and is responsible for the strength of induced electric dipole transitions. GdAlO_3_ with the *Pbnm* space group is appropriate for generating intense 4f electric dipole transitions, thereby enabling efficient luminescence. In recent years, trivalent rare-earth ions (RE^3+^) and transition-metal fluorescence in diverse host matrices have attracted significant attention due to their applications in persistent luminescent materials, photo-functional materials and luminescence thermometry.^7–10^ Doping GdAlO_3_ with transition metals and rare-earth ions is therefore of great interest for the development of advanced optical materials. Cr^3+^, in particular, is a transition-metal ion that acts as both a trapping and recombination center and has been widely studied in persistent luminescence research. Its unique properties can enhance the performance of imaging techniques, providing valuable insights into biological systems for *in vivo* bioimaging.^11,12^ However, the concentration of Cr^3+^ must be carefully optimized: a low concentration results in weak luminescence, whereas a high concentration leads to quenching, thereby reducing both the intensity and afterglow duration. A recent study by Jinan Xu *et al.*^13^ demonstrated that La_0.9898_Er_0.01_Sm_0.0002_Al_0.995_Cr_0.005_O_3_ exhibits long-term persistent luminescence at 1553 nm due to the Er^3+^: (^4^I_13/2_ → ^4^I_15/2_) transition, as well as at 734 nm, due to the Cr^3+^: (^2^E (^2^G) → ^4^A_2_ (^4^F)) transition. In ZnGa_2_O_4_ : Cr^3+^^14^ the persistent luminescence intensity increases with Cr^3+^ concentration up to 0.4–0.6%, after which concentration quenching reduces both intensity and lifetime. Moreover, in recent studies, the emission intensity of Cr^3+^ reaches its optimum at 0.5 mol% Cr^3+^ concentration and can be further enhanced by Li^+^ ion in Cr^3+^/Li^+^ co-doping ZnGa_2_O_4_ phosphor ^15^. The lifetimes of the ^4^T_2_ (^4^F) and ^2^E states of Cr^3+^ decrease with increasing concentrations of Cr^3+^ and Cr^3+^/Li^+^ ions.^15^ Similarly, a recent study reported by Ekta Rai *et al.*^16^ demonstrated that in Cr^3+^ and Eu^3+^ co-doped LaVO_4_, the emission intensity is optimal at 0.5 mol% Cr^3+^ and 1 mol% Eu^3+^ concentration. The emission intensity at 614 nm, corresponding to the ^5^D_0_ → ^7^F_2_ transition in the Eu^3+^ doped LaVO_4_ phosphor, reduces when Cr^3+^ ion is co-doped due to energy transfer between Cr^3+^ and Eu^3+^.^16^ This energy transfer was confirmed by the decrease of the lifetime of the ^5^D_0_ level of Eu^3+^ ions in Eu^3+^, Cr^3+^ co-doped LaVO_4_ phosphor.^16^ Understanding the energy levels of dopant ions, traps states, in GdAlO_3_ host and the energy transfer process between them is crucial for evaluating the suitability of material for optical applications such as LEDs, plant growth lighting, and *in vivo* optical imaging. The experimental origin of luminescence in GdAlO_3_ is studied by K Dhahri *et al*.^17^ The energy levels of Cr^3+^ combined with various trivalent lanthanides in GdAlO_3_ have been studied by Hongde Luo and Pieter Dorenbos ^18^. However, to the best of our knowledge, Er^3+^, Cr^3+^ Co-doped GdAlO_3_ has not yet been explored. Taking this into account, the present work reports for the first time the synthesis and investigation of a Er^3+^, Cr^3+^ co-doped GdAlO_3_. This study aims to elucidate the energy transfer process occurring between Er^3+^, Cr^3+^, and traps states (intrinsic defects). Furthermore, based on both experimental results and theoretical optical considerations, we propose a detailed mechanism for the energy transfer involving Er^3+^, Cr^3+^, and the trap states.

## 2. Experimental procedures (synthesis and characterization)

The GdAlO_3_, Gd_0.99_Er_0.01_AlO_3_ and Gd_0.99_Er_0.01_Al_0.995_Cr_0.05_O_3_ samples were prepared using a conventional solid-state reaction method. Gd_2_O_3_ (99%), Al_2_O_3_ (99%), Cr_2_O_3_ (99%), and Er_2_O_3_ (99%) were used as starting raw materials in stoichiometric amounts. The precursor materials were ground into fine powders using an agate mortar. The powders were initially annealed at 700 °C and then reground, pestled, and gradually heated to 1200 °C in an alumina crucible, where they were sintered for four hours. Finally, the powders were pressed into pellets with an 8 mm diameter. Several techniques were employed to characterize the physical and structural properties of the compounds. The phase compositions of Gd_0.99_Er_0.01_AlO_3_ and Gd_0.99_Er_0.01_Al_0.995_Cr_0.05_O_3_ were identified by X-ray diffraction (XRD) measurements using a Siemens D5000 X-ray powder diffractometer utilizing CuK_α_ radiation (*λ* = 1.5406 Å) over a range of 20°–100°, with a step size of 0.02°. The powder morphology and chemical homogeneity were studied by scanning electron microscopy (SEM) using a TESCAN VEGA3 SBH instrument equipped with an energy dispersive microscopy (EDS) detector. Raman spectra were recorded in the range of 50–1100 cm^−1^ using a Horiba LabRam HR Evolution micro-Raman confocal system, with wavelength laser excitations at *λ* = 532 nm, 633 nm, and 785 nm. Absorption and reflectance spectra were recorded using a (SHIMADZU, UV-3101PC) UV–vis–IR spectrophotometer. Photoluminescence emission (PL) and excitation (PLE) measurements were recorded using a Horiba–Jobin–Yvon Fluorolog 322 spectrometer in time-resolved mode, using a pulsed lamp with a 0.05 ms post-flash delay.

## 3. Results and discussions

### 3.1. Structural analysis

#### 3.1.1. Crystal structure and X-ray diffraction patterns

**Fig. 1:**
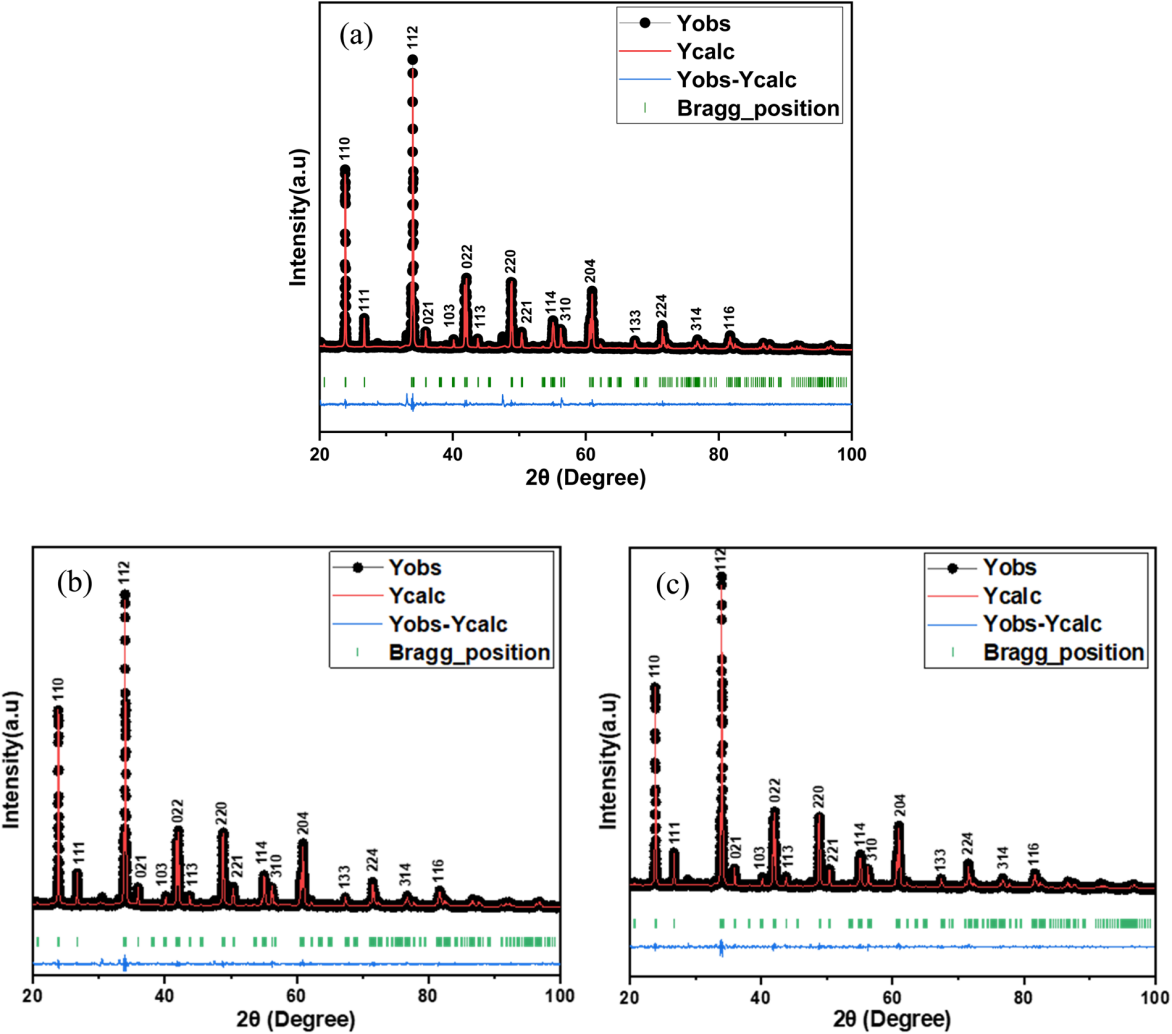
Rietveld refinement of X-ray diffraction pattern of (a) GdAlO_3_ (b) Gd_0.99_Er_0.01_AlO_3_ and (c) Gd_0.99_Er_0.01_Al_0.995_Cr_0.05_O_3._

**Fig. 2:**
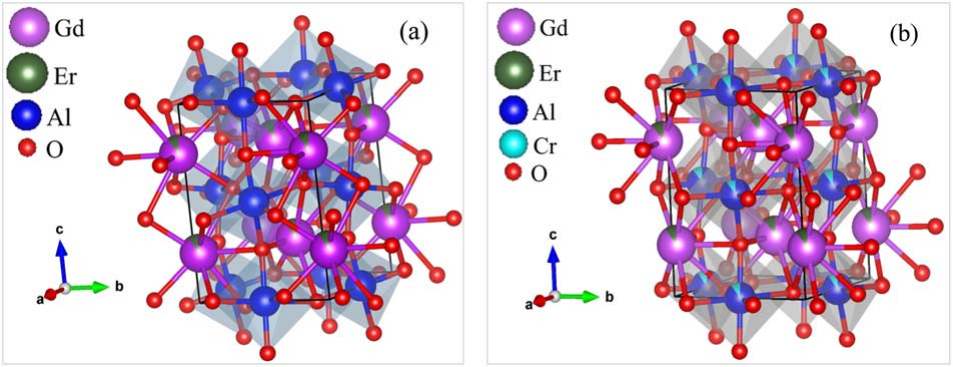
The Crystal structure using VESTA software for (a) Gd_0.99_Er_0.01_AlO_3_ and (b) Gd_0.99_Er_0.01_Al_0.995_Cr_0.05_O_3_ compounds.

**Fig. 3:**
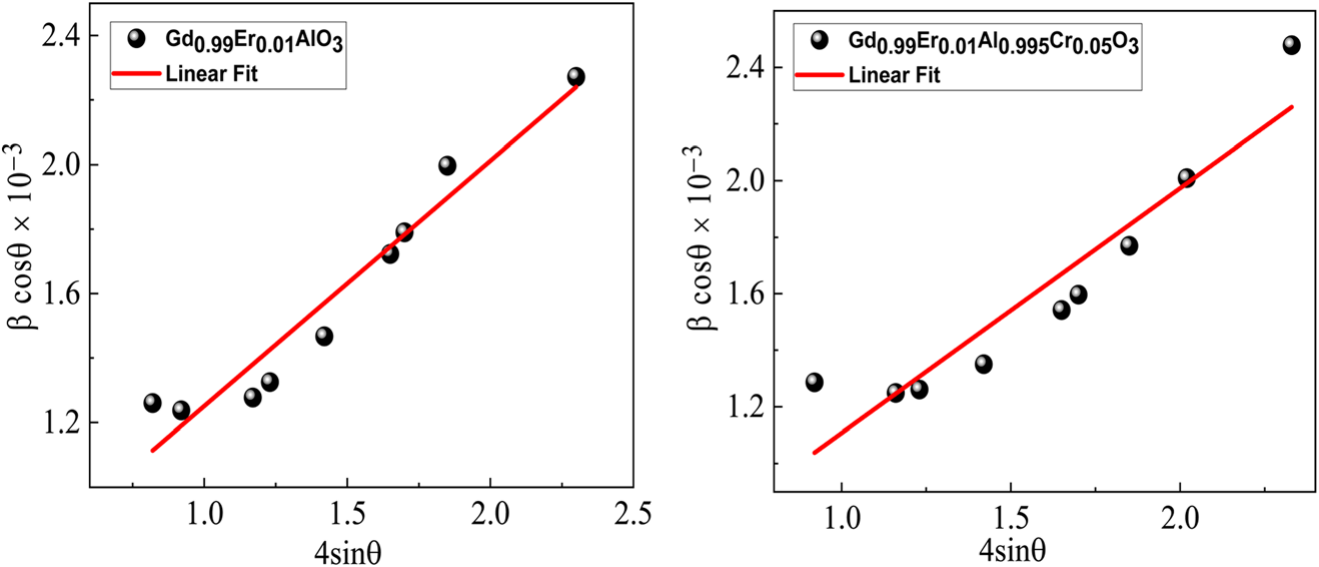
W–H plots of Gd_0.99_Er_0.01_AlO_3_ and Gd_0.99_Er_0.01_Al_0.995_Cr_0.05_O_3_ compounds.

The XRD pattern analysis of the GdAlO_3_, Gd_0.99_Er_0.01_AlO_3_, and Gd_0.99_Er_0.01_Al_0.995_Cr_0.05_O_3_ samples was performed at room temperature and is shown in Fig. 1(a)–(c). The XRD data were refined using the Rietveld method in the Full Prof software suite.^19^ The diffraction peaks align closely with the crystal planes of the orthorhombic GdAlO_3_ structure all diffraction peaks are indexed according to the PDF card no. 46-0395.^20^ Refinement results indicate that all samples crystallize in the orthorhombic GdAlO_3_ structure phase with the *Pbnm* space group.^20^ The estimated Rietveld refinement parameters, including the goodness of fit (*χ*^2^), reliability factors (*R*-profile factor, *R*-Bragg factor, and *R*-crystallographic factor), lattice parameters, cell volumes (V), and interatomic distances, are listed in Table 1. The crystal structure of Gd_0.99_Er_0.01_Al_0.995_Cr_0.05_O_3_ and Gd_0.99_Er_0.01_AlO_3_ compounds using VESTA software is presented in Fig. 2. The average crystallite size (*D*_sc_) was estimated from the line broadening of the peak with the highest intensity associated with the plane (112), using the Debye–Scherrer formula.^21^1
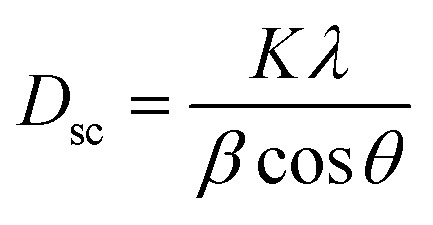
where *K* = 0.9 for spherical shape, *λ* is the wavelength of X-ray used, *β* is the full width at half maximum (FWHM) of the diffraction peak, and *λ* is the Bragg angle for the most intense peak. Furthermore, XRD peak broadening also has a contribution from the self-induced strain (*ε*) developed in crystallites during the growth that is effective in the nanoparticles.^22^ We additionally used the Williamson–Hall equation:^23^2
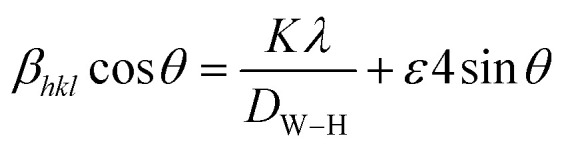
to determine the crystallite size and strain, taking into account the contribution of crystallites and strain to peak broadening. Where *K* is a constant (*K* = 0.9 for spherical shape), *λ* is the wavelength of the used X-ray, *β* is the full width at half maximum (FWHM) of the diffraction peak, and *ε* is the effective strain and *θ* is the Bragg angle for the most intense peak. Eqn (2) represents the Uniform Deformation Model (UDM), which assumes uniform strain in all crystallographic directions. The term (*β* cos *θ*) is plotted with respect to (4 sin *θ*) in Fig. 3 for the preferred orientation peaks (hkl) of Gd_0.99_Er_0.01_AlO_3_ and Gd_0.99_Er_0.01_Al_0.995_Cr_0.05_O_3_ samples showing that with the *y*-intercept and slope of the fitted line determining the crystallite size and related strain, respectively. The lattice strain observed is attributed to defects concentrated along the amorphous grain boundaries. These defects create a stress field within the grain boundary region, thereby inducing strain in the system.^24^Table 1 displays the crystallite size estimated from Debye Scherrer's formula and W–H plot as well as the related strain. The crystallite size increases considerably with Cr^3+^ doping. Cr^3+^ incorporation can increase both crystallite and particle size in certain oxide materials. When small amounts of Cr^3+^ ions substitute the cation Al^3+^ in the Gd_0_._99_AlEr_0_._01_O_3_ host lattice, they can induce lattice strain, modify the crystal growth process, and reduce the number of nucleation sites, leading to larger crystallites and particles. This effect is noticeable at low doping levels, as seen in Cr^3+^-doped gadolinium aluminum garnet and doped Mn_3_O_4_ systems.^25–28^ This fact explains the considerable increase of both crystallite and particle size of Gd_0.99_AlEr_0.01_O_3_ by Cr^3+^ doping with low concentrations. However, as the doping concentration increases further, excessive lattice distortion can inhibit growth, resulting in smaller crystallites and particles, a trend seen in several oxide systems.^25,26,28^

**Table 1:** Refined crystallographic parameters, average particle size and average strain value of GdAlO_3_, Gd_0.99_AlEr_0.01_O_3_ and Gd_0.99_Er_0.01_Al_0.995_Cr_0.05_O_3_ samples

Compounds	GdAlO_3_	Gd_0.99_AlEr_0.01_O_3_	Gd_0.99_Er_0.01_Al_0.995_Cr_0.05_O_3_
*a* (Å)	5.253(2)	5.253(8)	5.253(6)
*b* (Å)	5.302(5)	5.303(7)	5.302(5)
*c* (Å)	7.447(2)	7.448(6)	7.447(8)
*v* (Å^3^)	207.442(4)	207.552(1)	207.474(9)
*d*_(Gd–Gd)_	—	3.736(7)	3.804(8)
*d*_(Gd–Al)_	—	3.072(3)	3.266(8)
*χ* ^2^	1.853	1.389	1.46
*R*_P_ (%)	12.3	11.7	14.8
*R*_wp_ (%)	11.5	10.4	12.0
*R*_e_ (%)	8.43	8.81	9.93
*D*_sc_ (nm)	—	108.5(6)	111.0(2)
*D*_W–H_ (nm)	—	283.1(4)	574.3(8)
*D*_SEM_ (nm)	—	300	639
*ε*	—	0.00076(1)	0.00086(6)

#### 3.1.2. SEM and EDS analysis

**Fig. 4:**
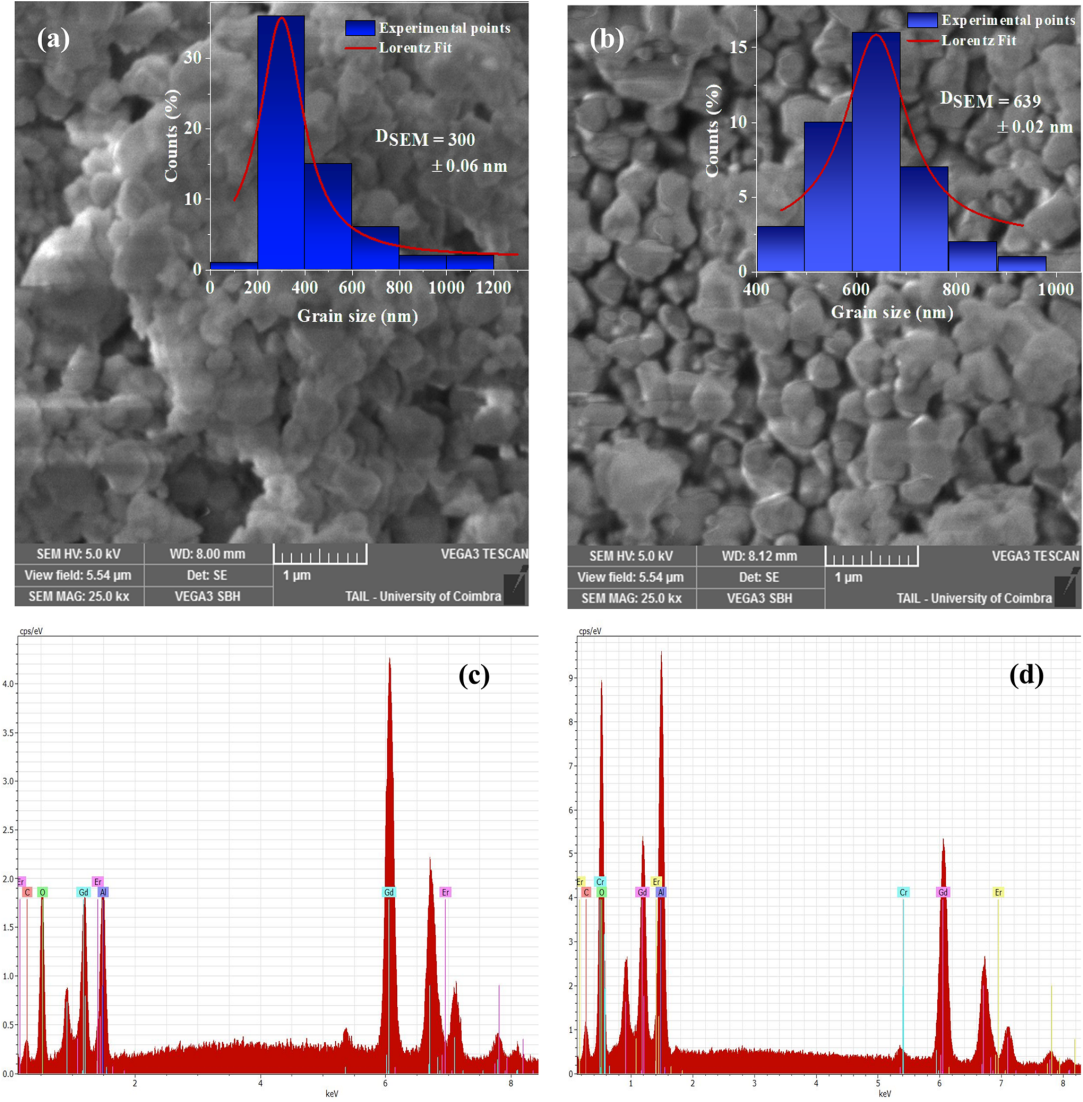
(a and b) SEM micrographs, with the inset showing the size distribution histogram for Gd_0.99_Er_0.01_AlO_3_ and Gd_0.99_Er_0.01_Al_0.995_Cr_0.05_O_3_ compounds, (c and d) spectra of chemical analysis for Gd_0.99_Er_0.01_AlO_3_ and Gd_0.99_Er_0.01_Al_0.995_Cr_0.05_O_3_ compounds.

The morphological characterization of Gd_0.99_Er_0.01_Al_0.995_Cr_0.05_O_3_ and Gd_0.99_Er_0.01_AlO_3_ compounds was carried out using scanning electron microscopy (SEM), as illustrated in Fig. 4(a) and (b), respectively. The SEM images show that the particles are approximately spherical. Due to the high surface energy of the nanoparticles, the synthesized samples exhibit noticeable aggregation at the annealing temperature.^29^ The grain size distribution, shown in the inset of Fig. 4(a) and (b), was analyzed using Image J software, and the resulting histograms were fitted to a Lorentzian function. The average grain size distribution revealed peaks around 300 nm for Gd_0.99_Er_0.01_AlO_3_ and 639 nm for Gd_0.99_Er_0.01_Al_0.995_Cr_0.05_O_3_ as presented in Fig. 4(a) and (b), and summarized in Table 1. Furthermore, energy dispersive spectra (EDS) were recorded for both samples, as illustrated in Fig. 4(c) and (d). The EDS spectra confirm the existence of the expected constituent elements: Gd, Er, Al, Cr, and O. These results further confirm the compositional purity of the synthesized compound.

#### 3.1.3. Raman spectra analysis of Gd_0.99_AlEr_0.0_O_3_ and Gd_0.99_Er_0.01_Al_0.995_Cr_0.05_O_3_ compounds

Raman spectroscopy is a powerful technique that extracts information on the development of the desired phase, detecting impurities, and identifying structural defects by examining Raman active phonon modes. The Raman spectra of GdAlO_3_ in the orthorhombic (*Pbnm*) perovskite structure have been studied both theoretically and experimentally by Anastasia Chopelas.^30^ For GdAlO_3_ with the orthorhombic (*Pbnm*) structure, group theory predicts the following optical modes in the Brillouin zone center.^30^

**Fig. 5:**
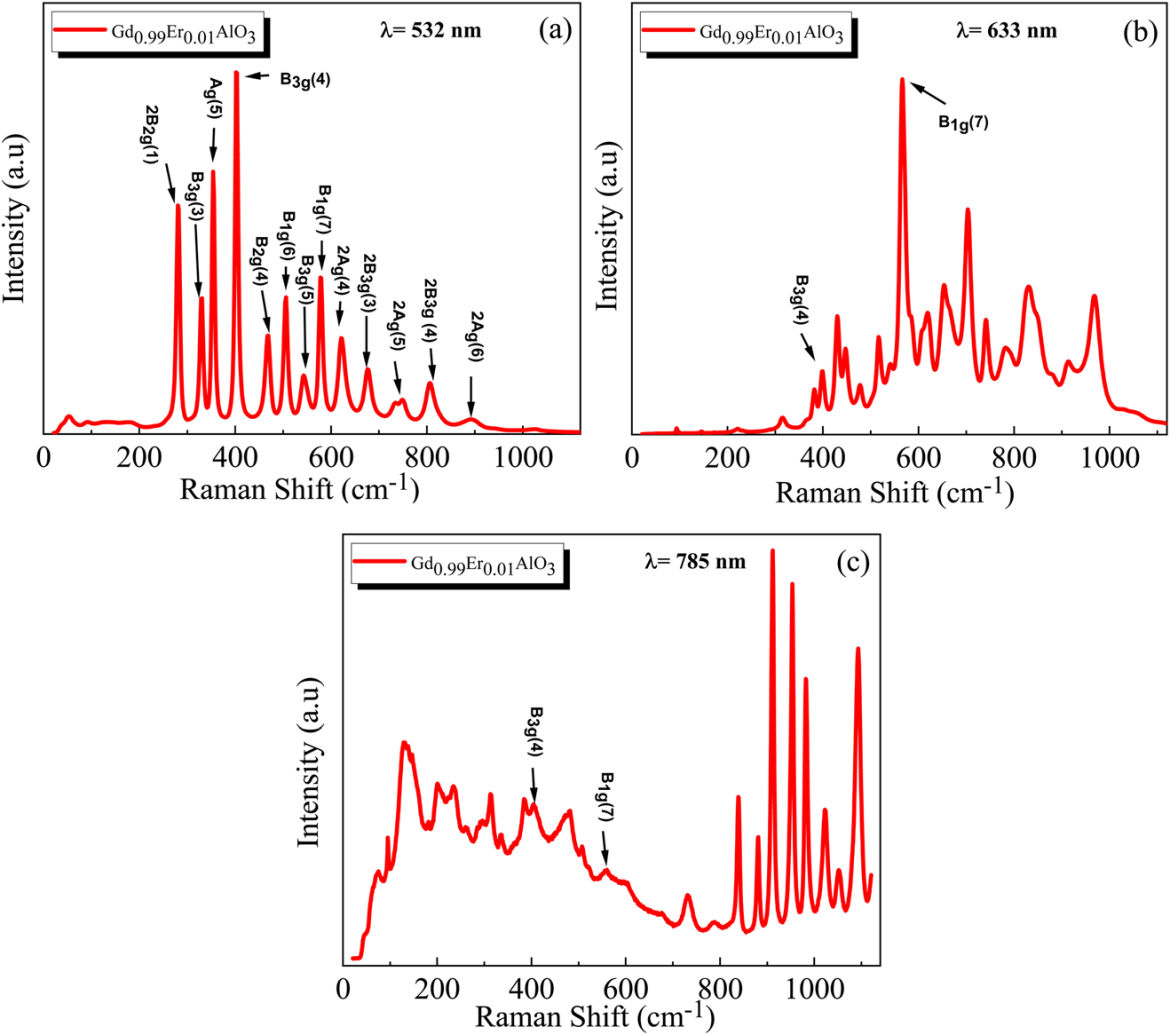
Raman spectra of Gd_0.99_AlEr_0.01_O_3_ under different excitation wavelengths: (a) 532 nm, (b) 633 nm, and (c) 785 nm.

**Fig. 6:**
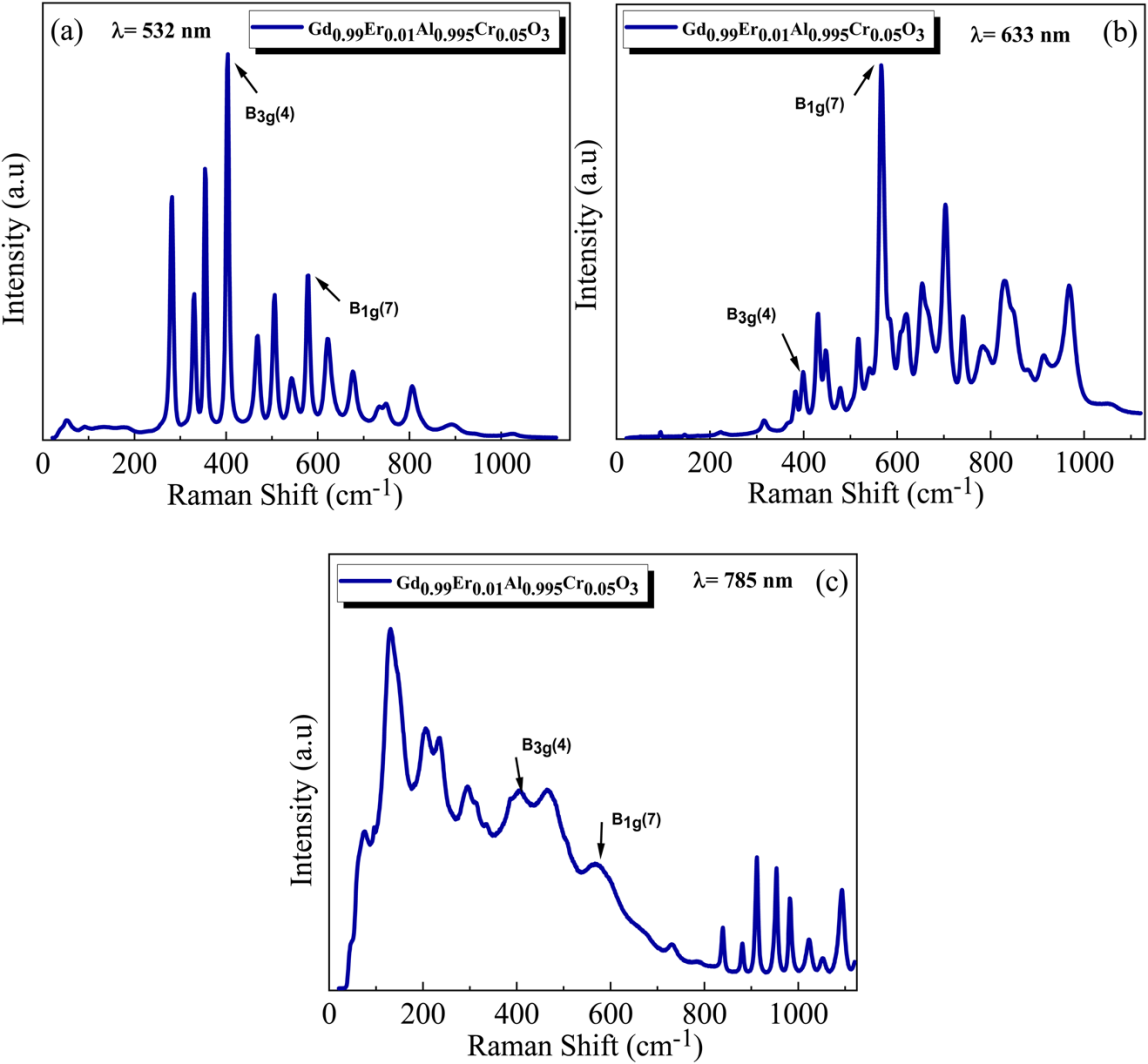
Raman spectra of Gd_0.99_Er_0.01_Al_0.995_Cr_0.05_O_3_ under different excitation wavelengths: (a) 532 nm, (b) 633 nm, and (c) 785 nm.

3 *Γ*′ = 7Ag® + 5B_1g_® + 7B_2g_® + 5B_3g_® + 8A_u_(1) + 7B_1u_® + 9B_2u_(IR) + 9B_3u_(IR)

where *R* and IR denote, respectively, their Raman and infrared spectral activity. Fig. 5 and 6 display the Raman spectra of Gd_0.99_Er_0.01_AlO_3_ and Gd_0.99_Er_0.01_Al_0.995_Cr_0.05_O_3_ respectively, recorded using excitation wavelengths of 532 nm, 633 nm, and 785 nm. By comparing the spectra obtained with various excitation wavelengths, it is possible to distinguish between Raman scattering and luminescence based on their distinct natures: bands with fixed locations are true Raman bands, whereas bands that shift in position are associated with luminescence.^31^ By extending the collection range to 1100 cm^−1^, our measurement revealed several intense and clearly non-vibrational extra bands above 579 cm^−1^. These bands were attributed to fluorescence as they resemble the characteristic f–f transitions of trivalent lanthanide ions. The appearance of resonance Raman, resonance fluorescence and relaxed fluorescence can be attributed to the excitation energies of the wavelength's excitation 532 nm, 633 nm, and 785 nm which are resonant with the transitions ^4^I_15/2_ → ^4^S_3/2_, ^4^I_15/2_ → ^4^F_9/2_, ^4^I_15/2_ → ^4^I_9/2_ of Er^3+^, respectively.^32^ Raman spectra of Gd_0.99_Er_0.01_AlO_3_ and Gd_0.99_Er_0.01_Al_0.995_Cr_0.05_O_3_ collected with 532 nm laser excitation are shown in Fig. 5 (a) and 6(a). They present prominent peaks at 330 cm^−1^, 355 cm^−1^, 403 cm^−1^, 468 cm^−1^, 507 cm^−1^, 545 cm^−1^, 579 cm^−1^ assigned respectively to Resonance Raman and resonance fluorescence associated to the vibrations modes B_3g_(3), Ag(5), B_3g_ (4), B_2g_ (4), B_1g_(6), B_3g_(5), B_1g_(7).^30^ The peaks at 281 cm^−1^, 625 cm^−1^, 677 cm^−1^, 734 cm^−1^, 807 cm^−1^, 892 cm^−1^, 947 cm^−1^, 1026 cm^−1^ are assigned to the vibration modes 2B_2g_(1), 2A_g_(4), 2B_3g_(3), 2A_g_(5), 2B_3g_(4), 2A_g_(6), 2B_2g_ (4), 2B_1g_(6)^30^ related to relaxed fluorescence. The bands of Raman spectra of Gd_0.99_Er_0.01_AlO_3_ and Gd_0.99_Er_0.01_Al_0.995_Cr_0.05_O_3_ recorded with 785 nm laser excitation in the vicinity of 1000 cm^−1^ are clearly due to relaxed fluorescence. Some vibration modes such Ag(3) at 235 cm^−1^ (ref. 30) occur only under 785 nm excitation, the vibrations mode B_1g_(3) at 220 cm^−1^ appear only under 633 nm excitation, whereas the vibrations mode at Ag(4) at 314 cm^−1^ appear only under 633 nm excitation. The bands at 403 cm^−1^ and 579 cm^−1^ due to B_3g_(4) and B_1g_(7) vibration modes are true Raman bands since they have fixed locations and do not depend on the wavelength excitation. They appear under all the wavelengths excitations of 532 nm, 633 nm, and 785 nm.

### 3.2. Optical properties

#### 3.2.1. Absorbance, reflectance spectra and band gap determination

**Fig. 7:**
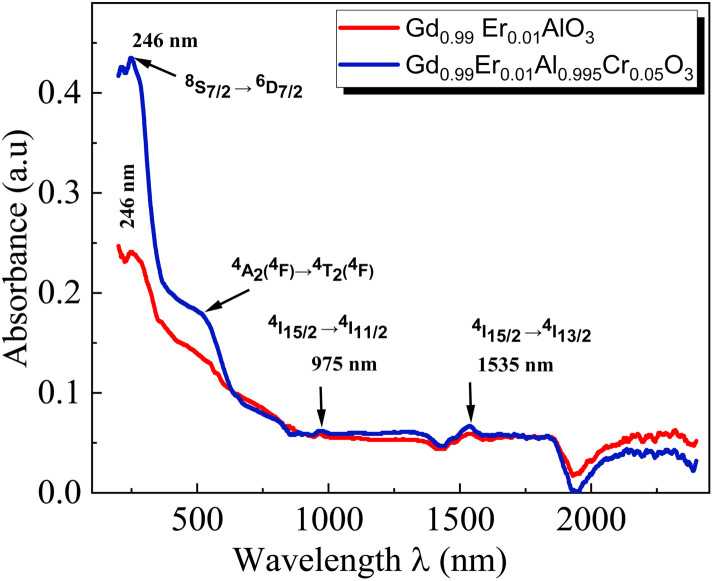
Absorbance spectra of Gd_0.99_Er_0.01_AlO_3_ and Gd_0.99_Er_0.01_Al_0.995_Cr_0.05_O_3_ samples at room temperature in the wavelength range 200–2500 nm.

The wavelength–dependent absorbance spectra of Gd_0.99_Er_0.0_AlO_3_ and Gd_0.99_Er_0.01_Al_0.995_Cr_0.05_O_3_, recorded in the 200–2500 nm range, are illustrated in Fig. 7. Both spectra exhibit an intense absorption band at 246 nm and two weaker bands at 975 nm and 1535 nm. With Cr^3+^ co-doping, an additional broad band appears at 565 nm. The absorption band at 246 nm is assigned to ^8^S_7/2_ → ^6^D_7/2_ transition of Gd^3+^ ions,^33^ whereas the peaks at 975 nm and 1535 nm correspond to the ^4^I_15/2_ →^4^I_11/2_ and ^4^I_15/2_ →^4^I_13/2_, transitions of Er^3+^ ions,^32^ respectively. The additional broad band in the absorbance spectrum of Gd_0.99_Er_0.01_Al_0.995_Cr_0.05_O_3_ at 565 nm is assigned to the ^4^A_2_ (^4^F) → ^4^T_2_ (^4^F) transition of Cr^3+^ ions. The band gap energy of the samples needs to be correctly determined in order to predict semiconductor optical properties. The derivation of absorption spectrum fitting (DASF), a precise method developed by Souri and Tahan,^34^ was used to ascertain the band gap's value and nature. The main advantage of this method is that it does not require any presumption of the nature of the optical transition and linear extrapolation. The absorption coefficient can be expressed as a function of the optical gap and the energy of photons as follows:^35,36^

4 *α*(*ν*)*hν* = *B*(*hν* − *E*_g_)^*m*^

By rewriting the eqn (1) as a function of the wavelength (*λ*): eqn (4) become:5
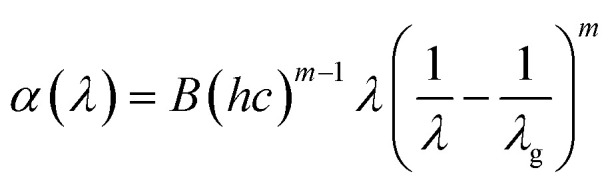
where *α*(*λ*), is the absorption coefficient defined by the Beer–Lambert's law as:6
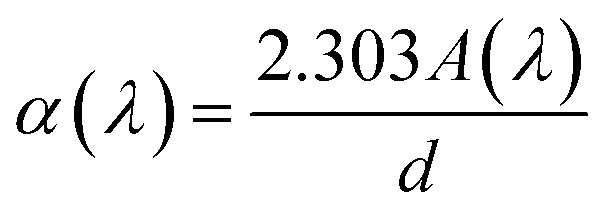
where *d* and *A* as film thickness and film absorbance. Using (5) and (6); the absorbance can be rewritten as: 7
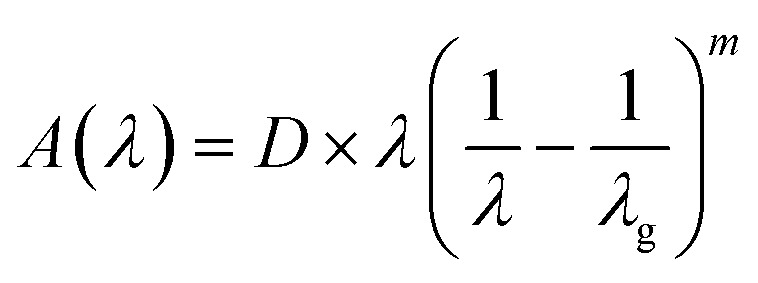
where: 
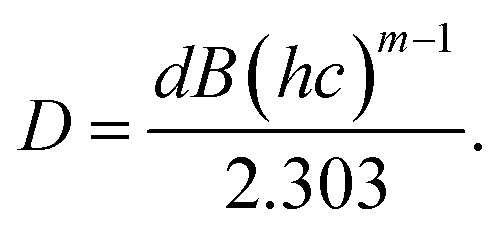


According to eqn (7) we have:8
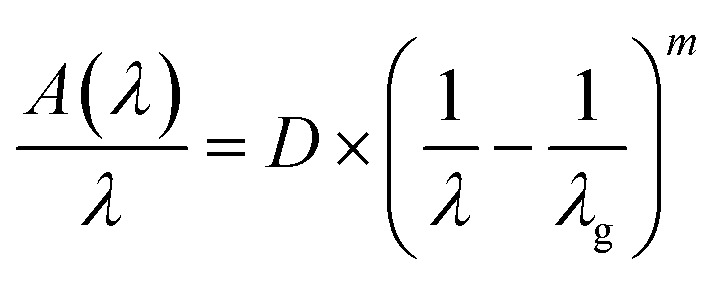


Eqn (8) can be reformulated as follows:9
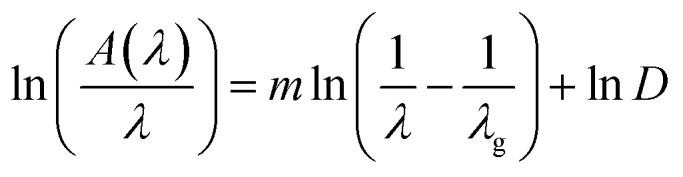


By differentiating 
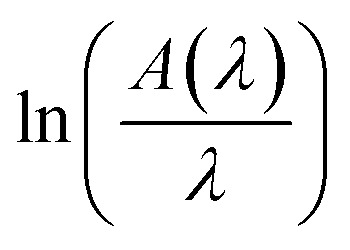
 with respect to 
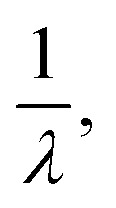
 we obtained the following equation:10
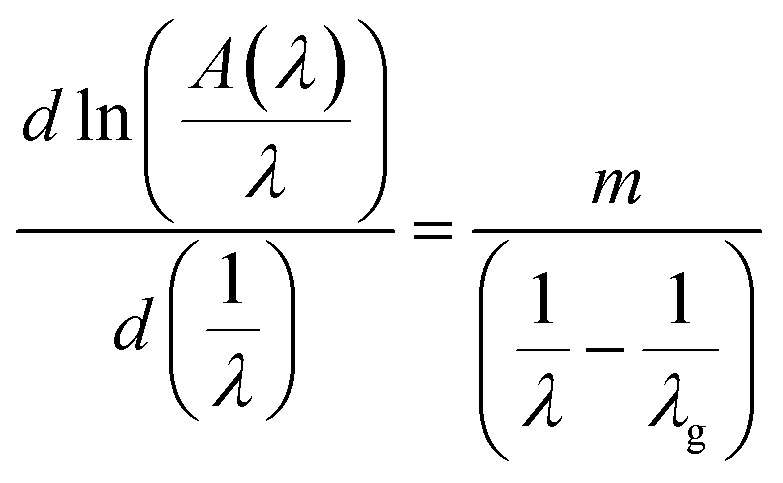


The band gap's value can be determined using the following expression for absorbance.^37^11
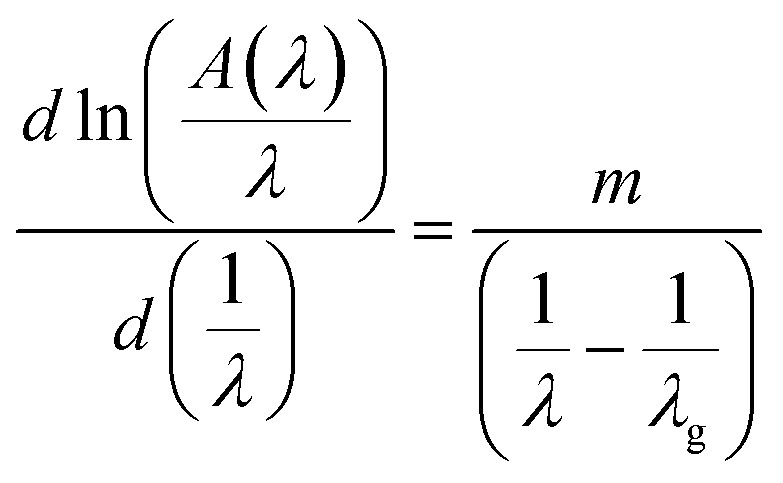


**Fig. 8:**
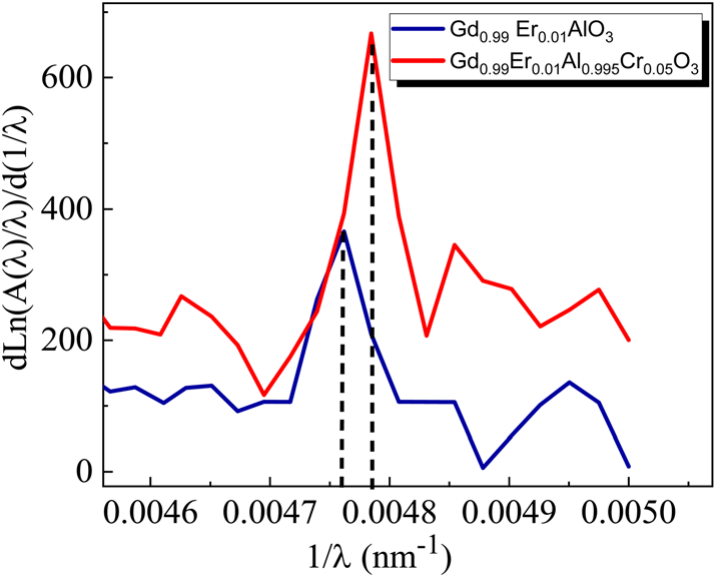
The variation of 
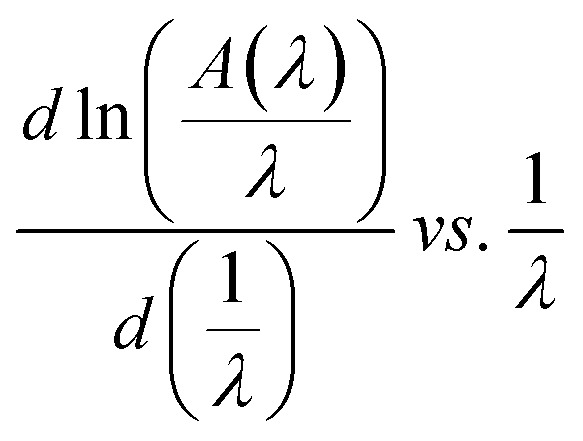
 for Gd_0.99_AlEr_0.01_O_3_ and Gd_0.99_Er_0.01_Al_0.995_Cr_0.05_O_3_ samples.

**Fig. 9:**
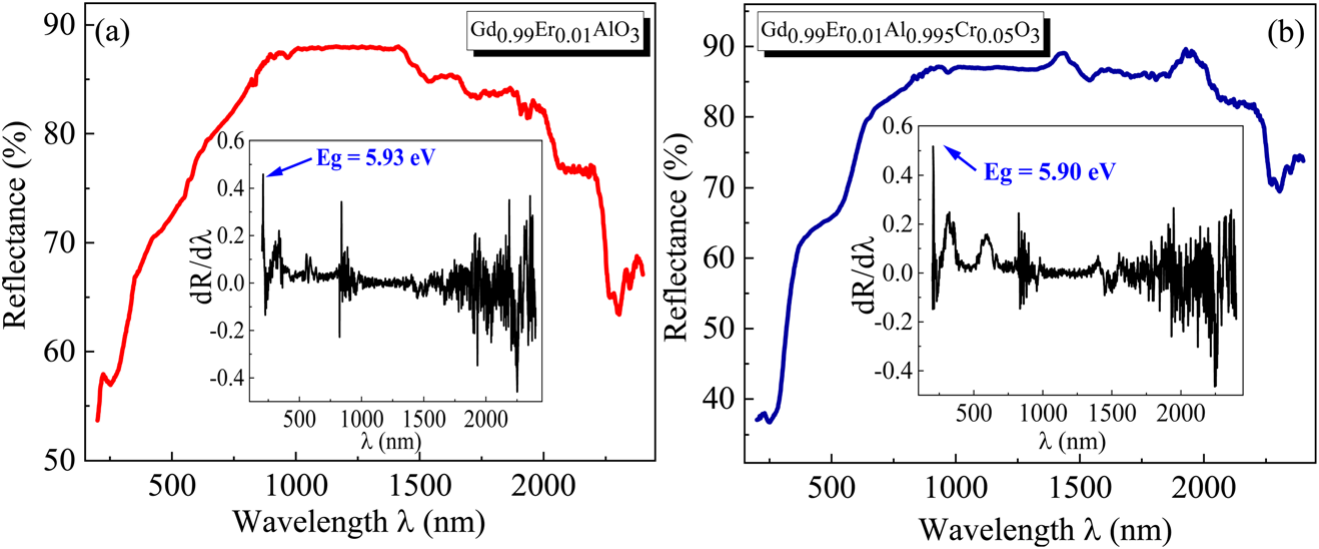
The room temperature reflectance spectrum *R*(*λ*) of (a) Gd_0.99_AlEr_0.01_O_3_ and (b) Gd_0.99_Er_0.01_Al_0.995_Cr_0.05_O_3_. The inset shows the evolution of d*R*/d*λ* with *λ*.

*A*(*λ*), *λ*, and *λ*_g_ are, respectively, absorbance, the incident wavelength, and the wavelength corresponding to the band gap energy. *m* and *D* are constants. The plot left side of eqn (11)
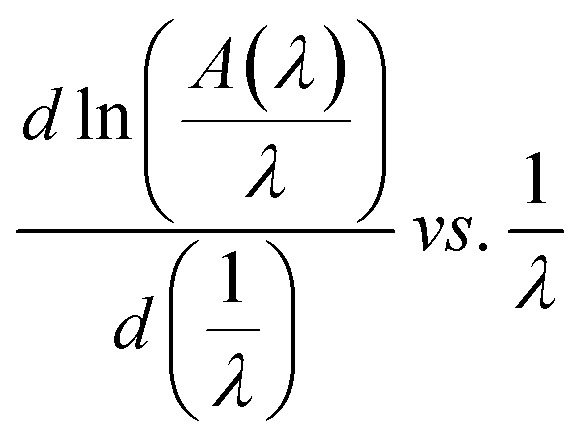
 for Gd_0.99_AlEr_0.01_O_3_ and Gd_0.99_Er_0.01_Al_0.995_Cr_0.05_O_3_ samples is shown in Fig. 8. The peak maxima can be used to determine the band gap energy, as seen in Fig. 8, at 
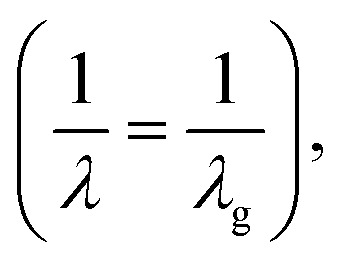
 the peak maximum discontinuity occurs. The optical band gap is computed as 
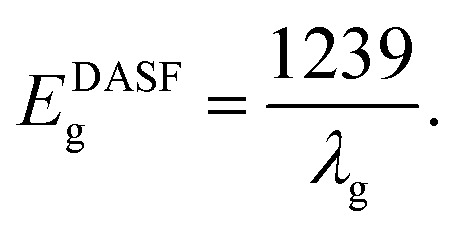
 Using the obtained λg, the resulting values are (5.93 ± 0.01) eV and (5.90 ± 0.01) eV for Gd_0.99_Er_0.01_AlO_3_ and Gd_0.99_Er_0.01_Al_0.995_Cr_0.05_O_3_ respectively. Marotti *et al.* showed that for direct band gap semiconductors, d*R*/d*λ* peaks close to *E*_g_, whereas for indirect band gap compounds, it approaches zero. Fig. 9 confirms the direct character of the optical band gap Gd_0.99_Er_0.01_AlO_3_ and Gd_0.99_Er_0.01_Al_0.995_Cr_0.05_O_3_, indicating that d*R*/d*λ* reaches a maximum at about 5.93 eV and 5.90 eV for Gd_0.99_Er_0.01_AlO_3_ and Gd_0.99_Er_0.01_Al_0.995_Cr_0.05_O_3_ samples. The optical band gap values determined *via* the derivation of absorption spectrum fitting (DASF) and the first derivative of reflectance, d*R*/d*λ*, are the same. This supports the correctness of the band gap energy values found. The decrease in the band gap of Gd_0.99_AlEr_0.01_O_3_ when doped with low concentration of Cr^3+^ ions introduces localized energy levels within the band gap which act as both deep electron and deep hole traps within the Gd_0.99_AlEr_0.01_O_3_ band gap. These traps are localized and do not merge with the conduction or valence bands at low doping levels. At low concentrations, these levels do not significantly alter the overall electronic structure or the positions of the conduction and valence bands.^18^ The increased hybridization Cr–O between the Cr-3d and O-2p orbitals due Cr^3+^ incorporation modifies slightly the top of the valence band which may shift upward (due to Cr 3d–O 2p interactions) or the bottom of the conduction band may shift downward.^38^ So, the band gap remains nearly unchanged.

#### 3.2.2. Photoluminescence (PL) and photoluminescence excitation (PLE) spectra

**Fig. 10:**
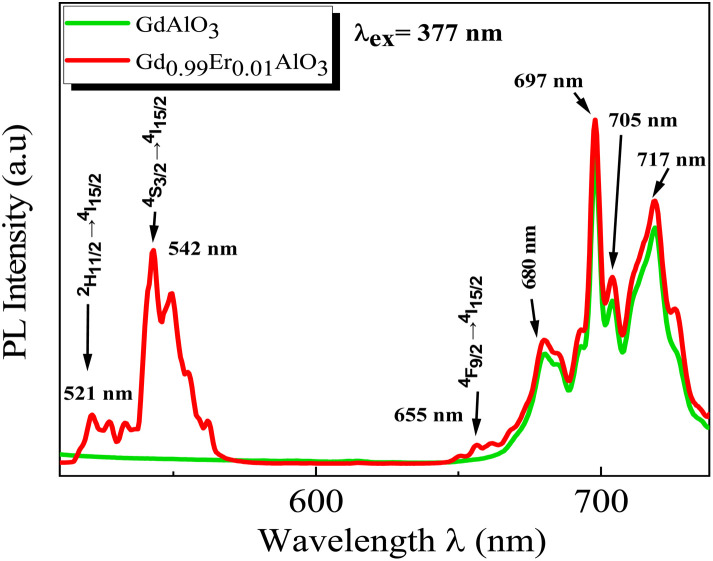
Room temperature PL emission spectra of the GdAlO_3_ and Gd_0.99_Er_0.01_AlO_3_ samples collected with excitation at 377 nm with flash-lamp and 0.05 ms delay after flash.

**Fig. 11:**
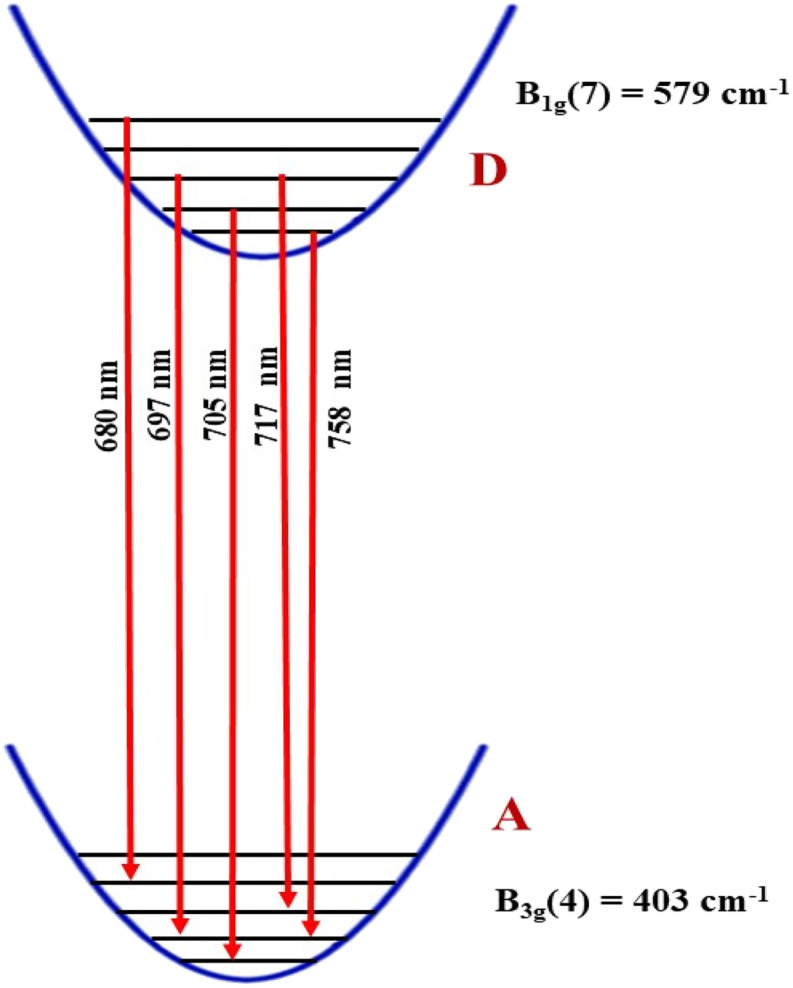
Configuration coordinate diagram showing the transition responsible for red emission lines which takes place between vibrational levels at (*T* = 300 K) in Gd_0.99_Er_0.01_AlO_3_ and Gd_0.99_Er_0.01_Al_0.995_Cr_0.05_O_3_.

**Fig. 12:**
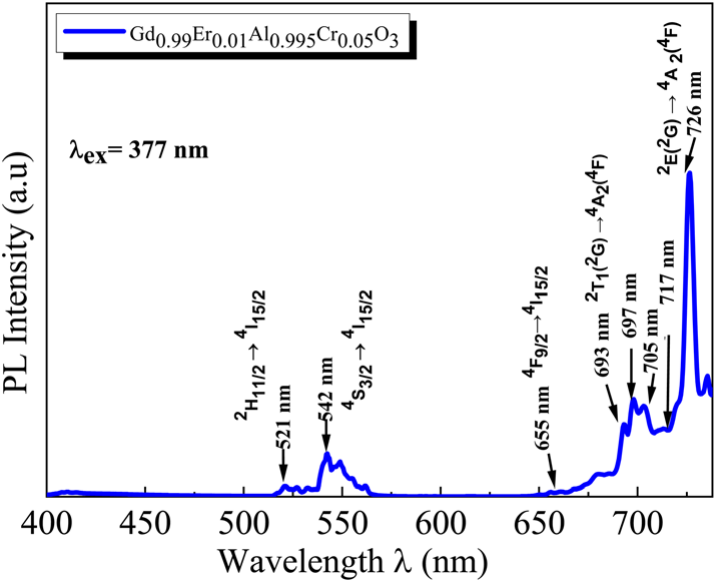
Room temperature PL spectrum of Gd_0.99_Er_0.01_Al_0.995_Cr_0.05_O_3_ in the wavelength range of 400–740 nm under 377 nm excitation.

**Fig. 13:**
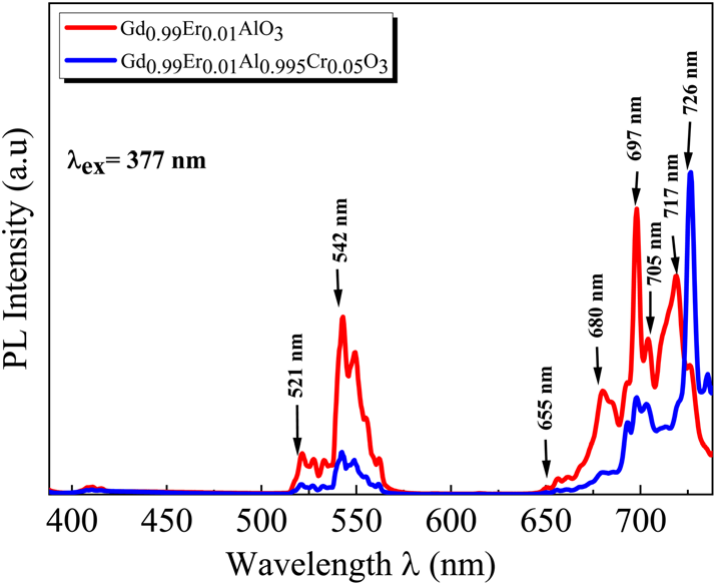
Room temperature PL spectra of Gd_0.99_Er_0.01_Al_0.995_Cr_0.05_O_3_ and Gd_0.99_Er_0.01_AlO_3_ samples under 377 nm excitation.

**Fig. 14:**
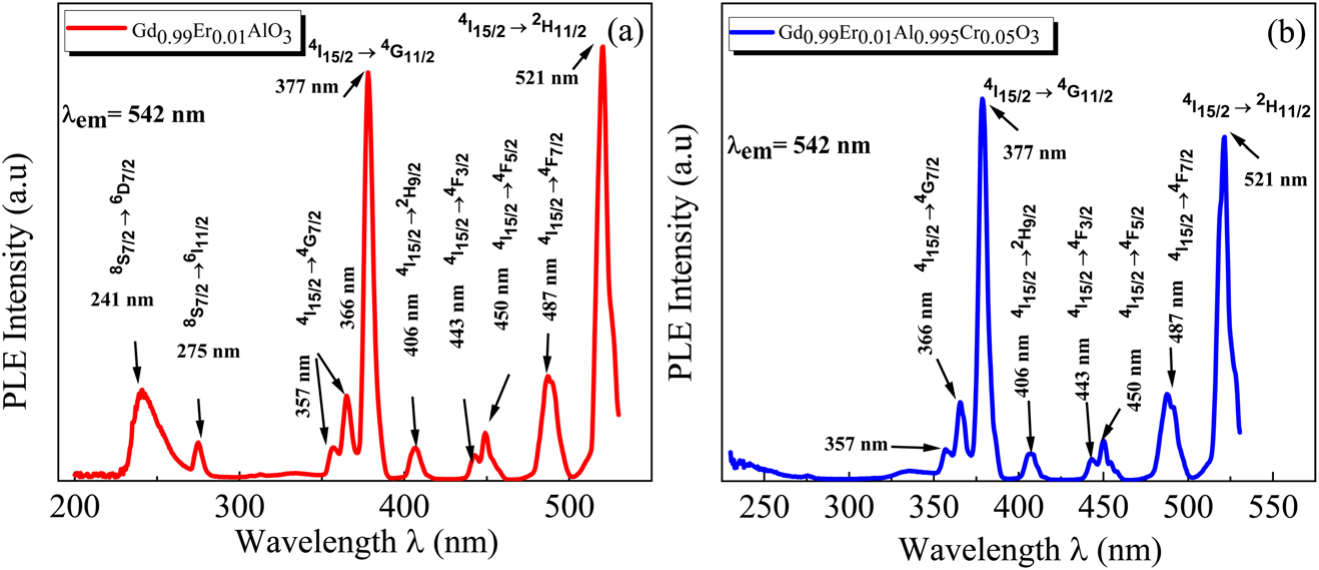
Room-temperature photoluminescence excitation (PLE) spectra monitored at 542 nm of (a) Gd_0.99_Er_0.01_AlO_3_ and (b) Gd_0.99_Er_0.01_Al_0.995_Cr_0.05_O_3_ (^4^S_3/2_ →^4^I_15/2_: Er^3+^).

The PL spectra of the GdAlO_3_ and Gd_0.99_Er_0.01_AlO_3_ samples, excited at 377 nm with a 0.05 ms delay after flash, in the wavelength range of 400–800 nm, are presented in Fig. 10. At room temperature, the PL spectrum of undoped GdAlO_3_ exhibits distinct sharp red emission lines at 680 nm (14 705 cm^−1^), 697 nm (14 347 cm^−1^), 705 nm (14 184 cm^−1^) 717 nm (13 947 cm^−1^), and 758 nm (13 192 cm^−1^). Upon Er^3+^ doping, these red emission lines persist, while additional bands appear in the wavelength ranges of 521–535 nm, 542–562 nm, and 655–657 nm, corresponding to the ^2^H_11/2_ → ^4^I_15/2_, ^4^S_3/2_ → ^4^I_15/2_ and ^4^F_9/2_ → ^4^I_15/2_ transitions of Er^3+^ ions, respectively.^32^ These bands are the result of intra-configurational 4f–4f transitions of Er^3+^ ions that appear within the 4f shell. According to the Laporte selection rule, 4f–4f transitions in rare-earth ions are parity-forbidden. The appearance of sharp spectral characteristics in Er^3+^ spectra could be explained by a non–central crystalline field's odd-order terms, which can create a coupling between odd and even states. Thus, resulting in mixed states of the 4fn with the first excited 4f^*n*−1^ 5d configuration, which mitigates Laporte's rule. This fact is responsible for the high intensity of induced electric dipole transitions observed in the PL and PLE spectra.^39^ The red lines at 680, 697, 717, 705 and 758 nm are superimposed in both GdAlO_3_ and Gd_0.99_Er_0.01_AlO_3_, indicating that Er^3+^ doping does not modify the structure of the sharp lines and their energy locations confirms that these sharp red lines originate from the host GdAlO_3_ matrix. The red lines cannot originate from intraconfigurational transitions of Gd^3+^ ions since the excitation wavelength is 377 nm and the first excited state is ^6^P_7/2_ at 314 nm. In order for 4f–4f transitions of Gd^3+^ to take place under 377 nm excitation, two photons of 377 nm absorption must happen. Since we used a pulsed lamp with low intensity rather than a high-power laser, this is not possible. Similar sharp red emission lines under 320 nm excitation were also reported in undoped GdAlO_3_ by Kh. Dhahri *et al.*^17^ Based on their findings, the red emission in GdAlO_3_ is attributed to the presence of oxygen vacancies, singly ionized Vo^+^. In CaGdAlO_4_-type layered perovskites,^40^ deep red luminescence (emission around 711 nm) under 338 nm excitation is attributed to oxygen defects, especially oxygen interstitials. These defects create localized energy states within the bandgap, enabling radiative recombination that results in red light emission when the material is excited by UV or visible light. The origin of red luminescence in undoped GdAlO_3_ is primarily linked to intrinsic crystal defects, specifically oxygen-related defects, rather than the presence of intentional dopants. In addition, under 532 nm excitation, undoped YAlO_3_ single crystals exhibit emission bands in the wavelength range 670–800 nm, including peaks at ∼688, 703, 715, 732, and 750 nm,^41^ similar to those observed in GdAlO_3_ (ref. 17) and to the red lines found in the present work. The PLE spectrum monitored at the emission wavelength 715 nm in YAlO_3_ shows the strong excitation bands at ∼320 nm and 315 nm.^41^ In the distorted perovskite structure of GdAlO_3_, cation vacancies such as Vo^+^ and Vo^++^ are the dominant intrinsic defects to neutralize the minor amount of Cr^3+^ and Er^3+^.^42^ Taking this into account, the photoluminescence (PL) process responsible for the red emission under 377 nm excitation can be described as follows: under excitation at 377 nm, electrons are excited from the valence band and subsequently trapped by intrinsic defect. These trapped electrons then relax and are captured by deep acceptor states associated with intrinsic defects. According to the configuration coordinate model, the resulting red emission peaks can be ascribed to electron transitions between donor and acceptor levels associated with vibrational modes B_3g_(4) and B_1g_(7) as shown in Fig. 11. With chrome co-doping, an additional intense red emission line appears at 726 nm, and a weak peak at 693 nm, as shown in the room-temperature PL spectrum of Gd_0.99_Er_0.01_Al_0.995_Cr_0.05_O_3_ in Fig. 12. The 726 nm emission corresponds to the ^2^E (^2^G) → ^4^A_2_(^4^F) transition of Cr^3+^ ions.^43^ The co-doping by chrome induces a significant decrease in the emission intensity of the Er^3+^ ions, a dramatic decrease in the emission line intensity at 680 nm, 697 nm, 717 nm, 705 nm. A low intensity peak at 693 nm occurs assigned to the transition from the fundamental state ^4^A_2_(^4^F) to the sublevel of ^2^T_1_(^2^G) split by spin–orbit coupling. An intense emission line emerges at 726 nm Fig. 13. Photoluminescence excitation (PLE) spectra of Gd_0.99_Er_0.01_Al_0.995_Cr_0.05_O_3_ and Gd_0.99_Er_0.01_AlO_3_ monitored at 542 nm are presented in Fig. 14a and b, respectively. The room-temperature PLE spectrum of Gd_0.99_Er_0.01_Al_0.995_Cr_0.05_O_3_ monitored at 542 nm, corresponding to an Er^3+^ transition, shows typical Er^3+^ excitation lines peaking at around (357 nm, 366 nm), 377 nm, 406 nm, 443 nm, 450 nm, 487 nm, and 521 nm. These lines are attributed to the following transitions: ^4^I_15/2_ → ^4^G_7/2_, ^4^I_15/2_ → ^4^G_11/2_, ^4^I_15/2_ → ^2^H_9/2_, ^4^I_15/2_ → ^4^F_3/2_, ^4^I_15/2_ → ^4^F_5/2_, ^4^I_15/2_ → ^4^F_7/2_, ^4^I_15/2_ → ^2^H_11/2_, respectively.^32^ The intensity *I* (*N*, *τ*) of the emission lines depends on both the population density *N* of the excited state and the radiative lifetime *τ* of the emitting level. The strongest excitation peak at 377 nm, corresponding to the transition^4^I_15/2_ → ^4^G_11/2_, can be justified by the fact that the transition ^4^I_15/2_ → ^4^G_11/2_ obeys the selection rule for an electronic–dipole transition in the context of Judd–Oeffelt theory.^39^

**Fig. 15:**
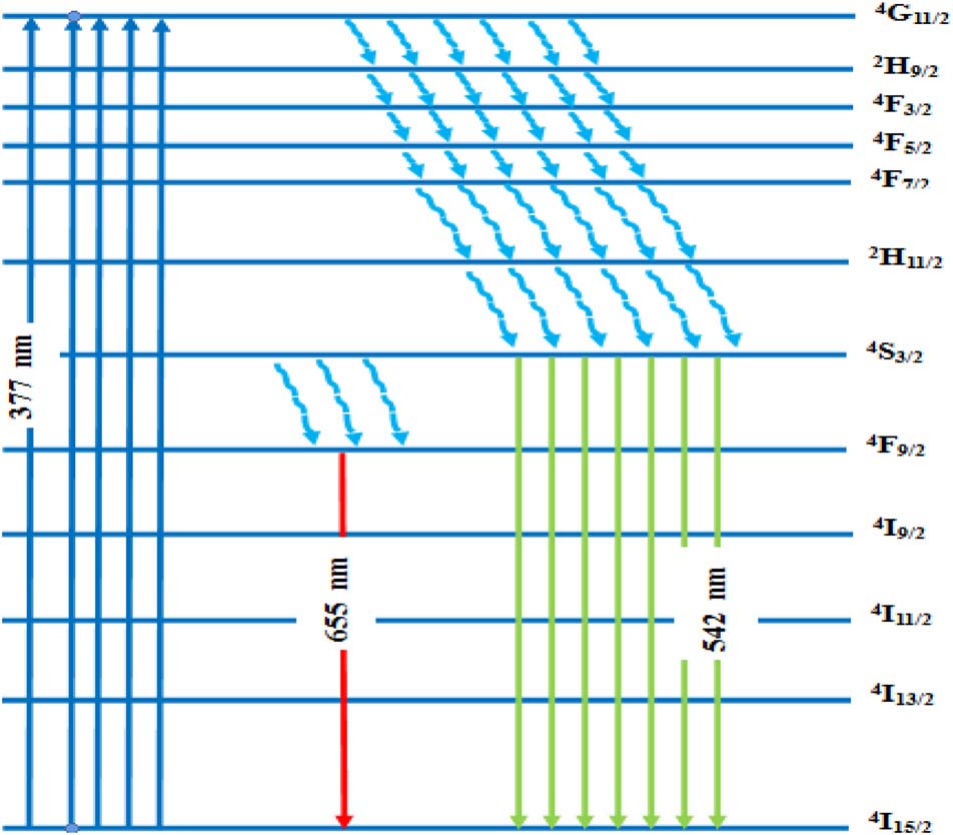
The Photoluminescence process at 542 nm under 377 nm in Gd_0.99_Er_0.01_AlO_3_ and Gd_0.99_Er_0.01_Al_0.995_Cr_0.05_O_3_.

The intensity of 4f–4f transitions of rare-earth elements within a host matrix can be described using the standard Judd–Ofelt (J–O) theory. According to this theory, the expressions for the electric dipolar line strength *S*_*JJ′*_^ED^ and electric dipolar oscillator strength *f*_cal_(*J*,*J*′) of transitions from the state |*S*, *L*, *J*> to the state |*S*′, *L*′, *J*′> are given by:^39^12
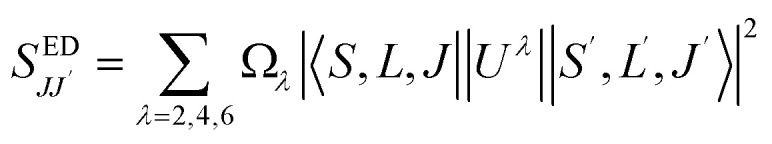
13
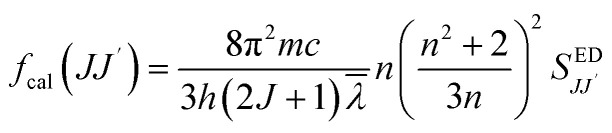
where *Ω*_*λ*_ are the Judd–Ofelt intensity parameters. The terms in brackets represent the doubly reduced matrix elements in intermediate coupling. *J* is the total angular momentum of the initial state, *h* is the Planck constant, *c* is the speed of light, *λ̄* is the mean wavelength corresponding to the specific absorption band of a transition |*S*, *L*, *J*> to the state|*S*′, *L*′, *J*′> and *n* is the refractive index of GdAlO_3_. Assuming that the host matrix has minimal influence on these values, we take this value as the value of Er^3+^ in aqueous solutions (aq), or Er^3+^ in LaF_3_ crystal as mentioned in ref. 44. The transitions from the ground state ^4^I_15/2_ to the excited states ^2^H_11/2_ and ^4^G_11/2_ are characterized by large reduced matrix elements of the unit tensor.^32^ Thus, they present a high population densities of Er^3+^ ions in these excited state ^4^G_11/2_ and ^2^H_11/2_. According to eqn (5) and (6), this may result in strong absorption exhibited in the PLE spectrum.^32^ The existence of a significant electric dipole transition ^4^I_15/2_ → ^4^G_11/2_ implies that Er^3+^ ions occupy non-centrosymmetric sites in the GdAlO_3_ lattice. The appearance of the emission line at 542 nm (^4^S_3/2_ → ^4^I_15/2_) transition under 377 nm excitation can be attributed to three factors: firstly, strong absorption to the ^4^G_11/2_ state since *λ*_ex_ = 377 nm is a resonant excitation, secondly the rate of multiphonon relaxation ^4^G_11/2_ → ^2^H_9/2_ → ^4^F_3/2_ → ^4^F_5/2_ → ^4^F_7/2_ → ^2^H_11/2_ → ^4^S_3/2_ exceeds the probability of radiative decay transitions to the ground state ^4^I_15/2_; finally, the high energy separation between the emitted level ^4^S_3/2_ and ^4^F_9/2_ level. However, the high intensity of the emission line at 542 nm under 521 nm excitation (^4^I_15/2_ → ^2^H_11/2_) can be attributed to the large reduced matrix elements of the unit tensor of this transition, leading to high cross-absorption to the ^2^H_11/2_ state. Moreover, the lowest energy separation (approximately 1000 cm^−1^) between the emitted level ^4^S_3/2_ and ^2^H_11/2_ levels increases the multiphonon relaxation. Hence, the multiphonon relaxation from ^2^H_11/2_ to ^4^S_3/2_ level is efficient, which induces the population of ^4^S_3/2_ level. The increase of the intensity of the 542 nm emission line (^4^S_3/2_ → ^4^I_15/2_) with decreasing wavelength excitation from 406 nm to 487 nm can be explained by the multi-phonon relaxation between the excited level and the emitting level ^4^S_3/2_, which is governed by the energy-gap law or phonon law.^45^ The multi-phonon relaxation rate (*W*_nr_) increases with decreasing energy separation between the excited levels and the emitting level ^4^S_3/2_. Hence, the emission at 542 nm increases with decreasing wavelength excitation from 406 nm (^4^I_15/2_ → ^2^H_9/2_) to 487 nm (^4^I_15/2_ →^4^F_7/2_). The energy separation between the ^4^S_3/2_ and ^4^F_9/2_ levels is approximately 3100 cm^−1^,^32^ requiring five phonons (579 cm^−1^ each) to bridge the gap. Therefore, the non-radiative relaxation from ^4^S_3/2_ to ^4^F_9/2_ highly inefficient. As a result, the red emission intensity at 655 nm is lower than the green emission intensity at 542 nm under 377 nm excitation in Gd_0.99_Er_0.01_AlO_3_ (Fig. 10). The Photoluminescence process at 542 nm under 377 nm excitation in Gd_0.99_Er_0.01_AlO_3_ is shown in Fig. 15.

**Fig. 16:**
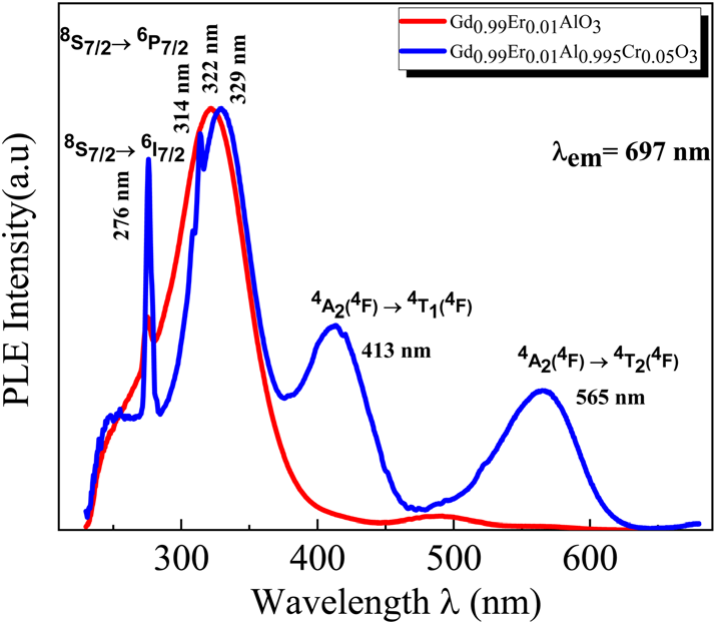
Room temperature PLE spectra in Gd_0.99_Er_0.01_AlO_3_ and Gd_0.99_Er_0.01_Al_0.995_Cr_0.05_O_3_ monitored at 697 nm.

The room-temperature PLE spectrum of Gd_0.99_Er_0.01_AlO_3_ monitored at 542 nm, shows the same characteristic excitation lines as those observed in Gd_0.99_Er_0.01_Al_0.995_Cr_0.05_O_3_ with two additional bands at 241 nm and 275 nm. These bands are attributed to the ^8^S_7/2_ → ^6^D_7/2_ and ^8^S_7/2_ → ^6^I_7/2_ transitions of Gd^3+^ ions, respectively.^46^ The disappearance of these emission bands in the Cr-doped sample (Gd_0.99_Er_0.01_Al_0.995_Cr_0.05_O_3_), under 542 nm monitoring, suggests the absence of energy transfer from Gd^3+^ to Er^3+^ in the presence of Cr^3+^. The room-temperature PLE spectrum of Gd_0.99_Er_0.01_AlO_3_ and Gd_0.99_Er_0.01_Al_0.995_Cr_0.05_O_3_ monitored at 697 nm are shown in (Fig. 16). The spectrum of Gd_0.99_Er_0.01_AlO_3_ exhibits two intense peaks at 322 nm and 275 nm, which assigned to the excitation of the electron from the valence band which trapped by the defect within the forbidden bandgap and the ^8^S_7/2_ → ^6^I_7/2_ transitions of Gd^3+^ ions, respectively. In contrast, the photoluminescence spectrum of Gd_0.99_Er_0.01_Al_0.995_Cr_0.05_O_3_ monitored at 697 nm reveals intense peaks at 329 nm and 276 nm, assigned to the excitation of the electron from the valence band which trapped by the defect and the ^8^S_7/2_ → ^6^I_7/2_ transitions of Gd^3+^ ions, respectively. Additionally, four extra bands are observed at 565 nm, 413 nm, 314 nm, and 241 nm. The broad bands around 565 nm and 413 nm are assigned to transitions from the ground state ^4^A_2_ (^4^F) to the excited states ^4^T_2_ (^4^F) and ^4^T_1_(^4^F) of Cr^3+^.^47^ The peaks at 314 nm and 241 nm are assigned to the (^8^S_7/2_ → ^6^P_7/2_:Gd^3+^) and (^8^S_7/2_ → ^6^D_7/2_: Gd^3+^) transitions, respectively.^46^

#### 3.2.3. Crystal field analysis and energy level schemes of Cr^3+^ ions in Gd_0.99_Er_0.01_Al_0.995_Cr_0.05_O_3_ nanoparticles

The energy levels of Cr^3+^ ions in Gd_0.99_Er_0.01_Al_0.995_Cr_0.05_O_3_ nanoparticles were calculated using the total Hamiltonian^48^

14 *H* = *H*_0_ + *H*_ee_(*B*, *C*) + *H*_Trees_(*α*) + *H*_CF_(*D*_q_) + *H*_SO_(*ξ*)

Eqn (14) describes the entire Hamiltonian *H*, where *H*_0_ is the configuration Hamiltonian term, and *H*_ee_(*B*, *C*), representing the electron–electron repulsion Hamiltonian. This term gives rise to the eight Russell–Saunders terms ^2S+1^L, including ^4^F, ^4^P, ^2^G, ^2^P, ^2^H, ^2^F, (^2^_a_D) and (^2^_b_D) for Cr^3+^ ions with 3 d^3^ configuration. *H*_CF_(*D*_q_) is the crystal field Hamiltonian, and *H*_SO_ (*ξ*) represents the spin–orbit coupling Hamiltonian. Using Racah algebraic techniques, the energy levels of the Russell–Saunders terms for the 3 d^3^ configuration are expressed in terms of Racah parameters *A*, *B*, and *C*, which depend on the double radial integrals *F* and *G*. The relative energies are those measured by optical spectroscopy. The quantity related to the *A* parameter is eliminated since it is the same for all the Russell–Saunders terms. Cr^3+^ ions (3 d^3^) are assumed to substitute Al^3+^ ions at the octahedral [AlO_6_] site in an intermediate crystal field (CF) strength. The basic function in the LS coupling scheme are expressed as:^49,50^

15 |*ψ*〉 = |*α*, SM_s_, LM〉

The crystal field energy levels can then be obtained by diagonalizing the entire Hamiltonian *H* = *H*_0_ +*H*_ee_ (*B*,*C*) + *H*_CF_ (*D*_q_) + *H*_SO_ (*ξ*). The crystal field Hamiltonian (*H*_CF_) in Wybourne notation is the spin–orbit hamiltonian and the Trees hamiltonian are expressed as follow:16
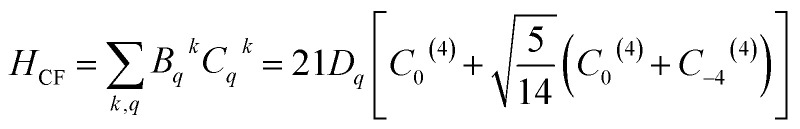
17
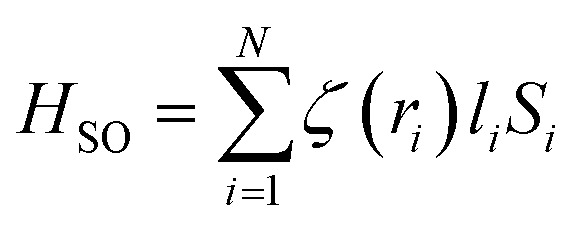

18 *H*_Trees_ = αL (*L*+ 1)

where *ξ*_3d_ is the spin–orbit coupling constant, *α* is the Trees parameter. The Racah and crystal field parameters *B*, *C*, and *D*_q_ are determined using the Newton–Raphson method by fitting the experimental energies levels to the theoretical ones ^4^A_2_ (^4^F) → ^4^T_2_ (^4^F) (565 nm), ^4^A_2_(^4^F) → ^4^T_1_(^4^F) (413 nm), and ^2^E(^2^G) →^4^A_2_(^4^F) (726 nm). The adjusted spin–orbit coupling *ξ*_3d_ and Trees parameter *α* are calculated as follows:19
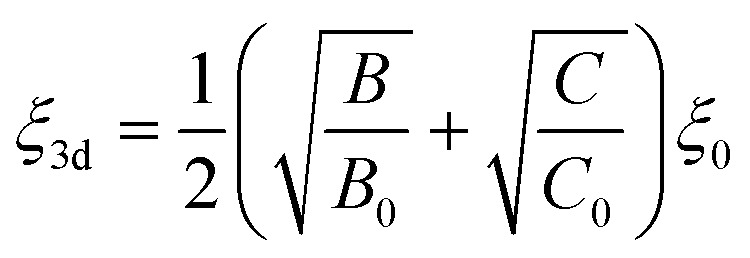
20
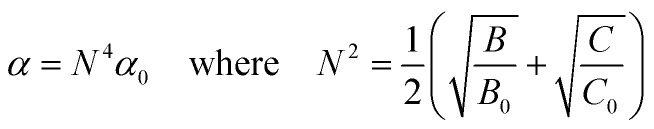


**Fig. 17:**
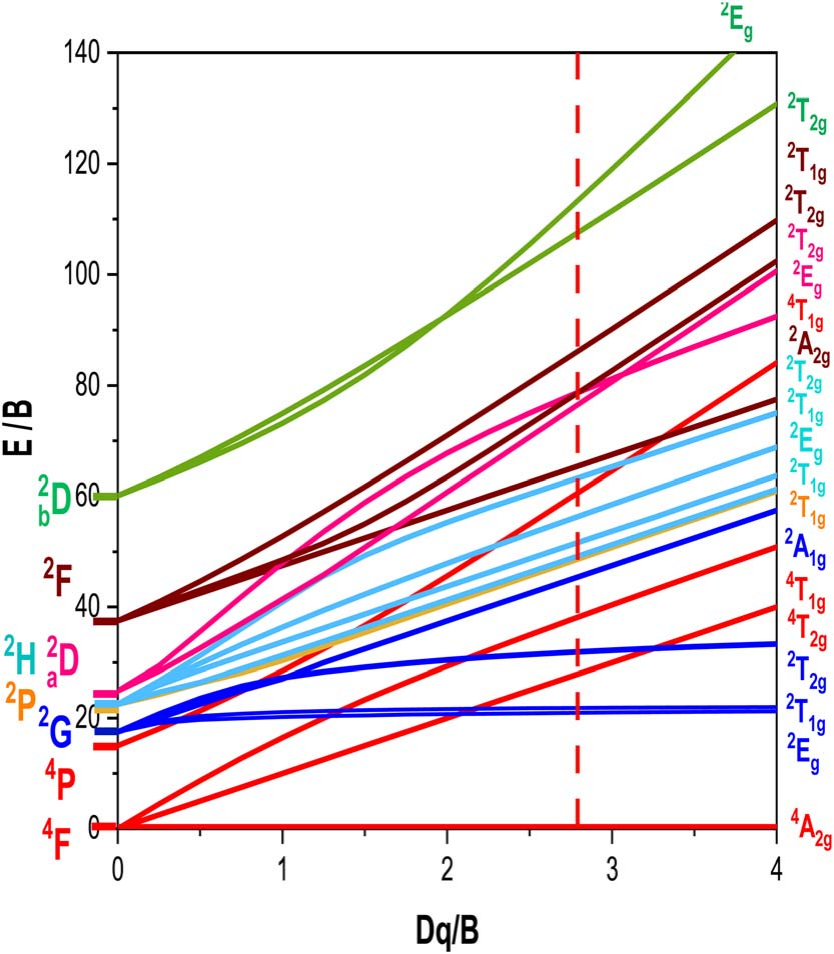
Tanabe–Sugano diagram for Cr^3+^ ions with *C*/*B* = 4.73. The vertical line at *D*_q_/*B* = 2.79 represents the energy levels identified for Cr^3+^in Gd_0.99_Er_0.01_Al_0.995_Cr_0.05_O_3_.

*B*_0_ = 918 cm^−1^, *C*_0_ = 4133 cm^−1^,^48^*ξ*_0_ = 275 cm^−1^ and *α*_0_ = 30 cm^−1^,^48^ which refer to the free ion parameters of Cr^3+^. The matrix elements of the crystal field, spin–orbit, and Trees Hamiltonians in the basics are provided by Y. Y. Yeung and C. Rudowicz.^50^ The full Hamiltonian matrix *H* (as defined in eqn (14)) was diagonalized to derive the energy levels as a function of the Racah parameters *B* and *C*, the crystal field parameter *D*_q_, and the spin–orbit coupling constant. This diagonalization was performed using unique code developed in our lab with the Maple program. The theoretical computed values are *B* = 635 cm^−1^, *C* = 3008 cm^−1^ and *D*_q_ = 1776 cm^−1^ with (*D*_q_/*B* = 2.79). The calculated parameters were used to calculate the energy levels at room temperature, as listed in Table 2. The Tanabe–Sugano diagram for Cr^3+^ ions in octahedral site symmetry, shown for the ratio *C*/*B* = 4.73 in Fig. 17, illustrates the overall behavior of Cr^3+^ energy levels in terms of *D*_q_/*B* relative to the local field intensity. The vertical line corresponds to the calculated *D*_q_/*B* value from our theoretical computation of Cr^3+^ levels in Gd_0_._99_Er_0_._01_Al_0_._995_Cr_0_._05_O_3_. It is well known that when *D*_q_/*B* < 2.3, Cr^3+^ ions experience a weak crystal field, resulting in broad-band emission. However, when *D*_q_/*B* > 2.3, the ions exhibit strong and narrow peak emission through the ^2^E(^2^G) → ^4^A_2_(^4^F) transitions.^51^ In our case, the calculated *D*_q_/*B* = 2.9, confirms that the energy of the ^2^E state is the lowest excited energy level. These results demonstrate that Cr^3+^ ions experience a strong crystal field, exhibiting sharp ^2^E(^2^G) → ^4^A_2_(^4^F) emission at 726 nm.

**Table 2:** Experimental and calculated energies levels (cm^−1^) of Cr^3+^ ion in octahedral symmetry in Gd_0.99_Er_0.01_Al_0.995_Cr_0.05_O_3_ nanoparticles

O_h_	*E*_obs_	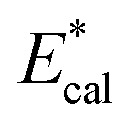 [this work]	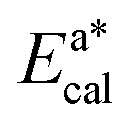 [this work]
^4^A_2g_(^4^F)	0	0	0
^2^E_g_(^2^G)	13 774	13 772	13 902 (4)
^2^T_1g_(^2^G)		14 312	14 434 (4)
			14 493 (2)
^4^T_2g_(^4^F)	17 760	17 760	17 666 (2)
			17 722 (4)
			17 832 (2)
			17 836 (4)
^2^T_2g_(^2^G)	—	20 906	20 974 (4)
			21 083 (2)
^4^T_1g_(^4^F)		24 276	24 165 (4)
			24 173 (2)
			24 185 (4)
			24 187(2)
^2^A_1g_(^2^G)	—	29 324	29 507 (2)
^2^T_1g_(^2^P)	—	31 382	31 582 (2)
			31 629 (4)
^2^T_1g_(^2^H)	—	31 712	31 803 (2)
			31 978 (4)
^2^E_g_(^2^H)	—	33 325	33 458 (2)
^2^T_1g_(^2^H)	—	36 266	36 324 (2)
			36 360 (4)
^4^T_1g_(^4^P)	—	38 528	38 388 (2)
			38 403 (4)
			38 503 (4)
			38 535 (2)
^2^T_2g_(^2^H)	—	40 671	40 574 (2)
			40 674(4)
^2^A_2g_(^2^F)	—	42 024	42 033 (2)
^2^T_2g_(^2^_a_D)	—	49 088	49 341 (2)
			49 451 (4)
^2^T_2g_(^2^F)	—	50 505	50 543 (2)
			50 652 (4)
^2^E_g_(^2^_a_D)	—	50 661	50 662(4)
^2^T_1g_(^2^F)	—	55 225	55 122 (2)
			55 246 (4)
^2^T_2g_(^2^_b_D)	—	69 140	69 008 (4)
			69 204(2)
^2^E_g_(^2^_b_D)	—	72 823	72 879 (4)

#### 3.2.4. Energy transfer process from Gd^3+^, Er^3+^ and oxygen defects to Cr^3+^ ions in Gd_0_._99_Er_0_._01_Al_0_._995_Cr_0_._05_O_3_

**Fig. 18:**
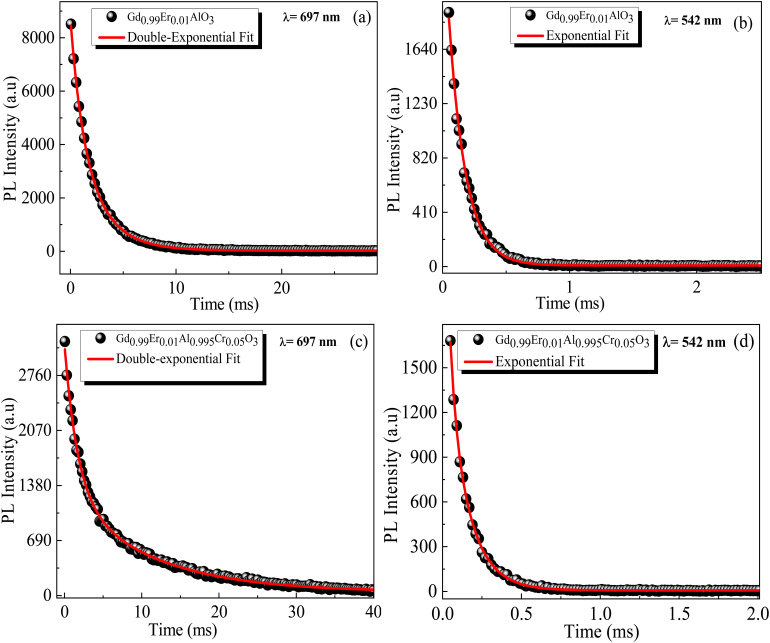
Decay PL curves under 377 nm excitation for Gd_0.99_Er_0.01_AlO_3_ at (a) 697 nm and (b) 542 nm, and for Gd_0.99_Er_0.01_Al_0.995_Cr_0.05_O_3_ at (c) 697 nm and (d) 542 nm.

The lack of Gd^3+^ transitions in the PLE spectrum of the Gd_0.99_Er_0.01_Al_0.995_Cr_0.05_O_3_ sample monitored at 542 nm proves the weak efficiency of energy transfer between Gd^3+^ and Er^3+^ ions. This can be attributed, first, to the shorter distance between Gd^3+^ and Cr^3+^ ions (*d*_Gd–Cr_ = 3.26683 Å, Table 1) compared to the distance between Gd^3+^ and Er^3+^ ions (*d*_Gd–Er_ = 3.80481 Å, Table 1) in Gd_0.99_Er_0.01_Al_0.995_Cr_0.05_O_3_. Second, the dominance of the higher energy transfer efficacy from Gd^3+^ to Cr^3+^ ions, which is explained by the higher trapping efficiency of the migrating excitation energy levels ^6^P_J_ and ^6^I_J_ of Gd^3+^ by Cr^3+^ activators in Gd_0_._99_Er_0_._01_Al_0_._995_Cr_0_._05_O_3_. Which is greater than by Er^3+^.^52^ This higher trapping efficiency is further explained by the spectral overlaps between the Cr^3+^ excitation band (^4^T_1_ (^4^P)) and the ^6^I_7/2_ level of Gd^3+^ ions.^52^ Notably, the peaks at 314 nm and 275 nm in the PLE spectrum of Gd_0.99_Er_0.01_Al_0.995_Cr_0.05_O_3_ monitored at 697 nm, present high intensities comparable to the peak at 329 nm. This experimental fact points out that under 314 nm and 275 nm, Gd^3+^ ions also act as donors of energy to the intrinsic defects *via* resonant phonon-assisted energy transfer. The decay PL curves at 697 nm and 542 nm under 377 nm excitation are shown in Fig. 18 (a) and (b) for Gd_0.99_Er_0.01_AlO_3_ and in Fig. 18 (c) and (d), for Gd_0.99_Er_0.01_Al_0.995_Cr_0.05_O_3_. The decay PL curves at 542 nm under *λ*_ex_ = 377 nm in Gd_0.99_Er_0.01_AlO_3_ is fitted to a monoexponential equation I(t) = Aexp(−*t*/*τ*) with fluorescence lifetimes 
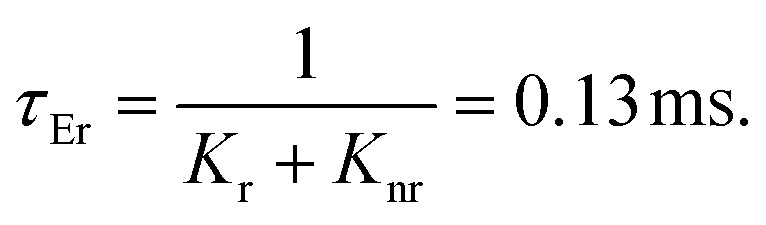
 The decay of the emission at 542 nm under 377 nm excitation in Gd_0.99_Er_0.01_Al_0.995_Cr_0.05_O_3_ is bi-exponential with lifetimes *τ*′ = 0.13 ms and 
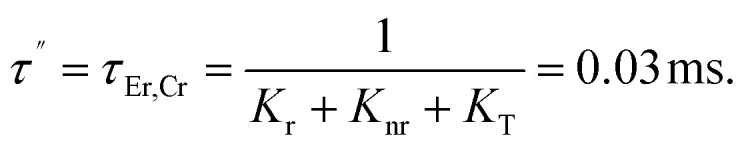
*K*_r_ is the emissive rate constant, *K*_nr_ is the non-radiative rate and *K*_T_ is the transfer rate constant. The decay curve shows two lifetimes: an unchanged longer time 0.13 ms (no transfer), a shorter time 0.03 ms (efficient transfer) which indicates an efficient energy transfer from a part of Er^3+^ ions to Cr^3+^ is taken place. The decay rate of Er^3+^ emission in Gd_0.99_Er_0.01_AlO_3_ and Gd_0.99_Er_0.01_Al_0.995_Cr_0.05_O_3_ samples as well as the ET efficiency from Er^3+^ to Cr^3+^ can be calculated using eqn (21)–(23),

21 *K*_r_ + *K*_nr_ = (*τ*_Er_)^−1^

22 *K*_r_ + K_nr_ + *K*_T_ = (*τ*_Er,Cr_)^−1^

23
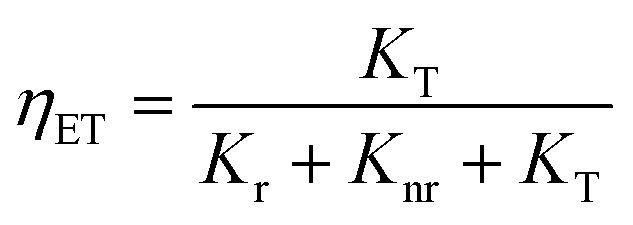


**Fig. 19:**
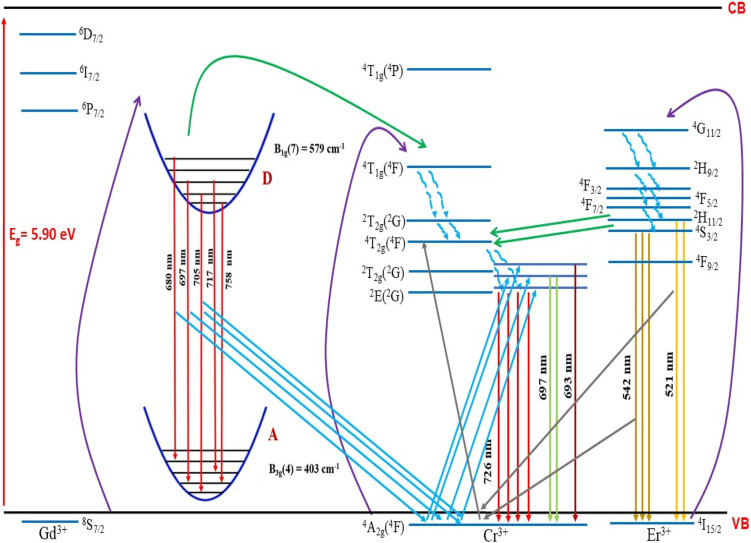
Energy level diagram and energy transfer mechanism in Gd_0.99_Er_0.01_Al_0.995_Cr_0.05_O_3_.

*τ*_Er_ and *τ*_Er,Cr_, are the life times of the Er^3+^ : ^4^S_3/2_ level in Gd_0.99_Er_0.01_AlO_3_ and Gd_0.99_Er_0.01_Al_0.995_Cr_0.05_O_3_ respectively. The ET efficiency (*η*_ET_) from Er^3+^ : ^4^S_3/2_ to the Cr^3+^: as indicated from the energy level diagram in Fig. 19 can be estimated to be 76%. Which indicate highly efficient energy transfer from Er^3+^ : ^4^S_3/2_ to the Cr^3+^ : ^4^T_2g_(^4^F). In rare-earth ion systems, energy transfer exchange interactions are generally negligible due to the small spatial extent of 4f orbitals, making multipolar mechanisms dominant. Specifically, in GdAlO_3_, efficient trapping of excitation energy by Cr^3+^ and rare earth ions like Er^3+^ is attributed to multipolar interactions when there is spectral overlap, while exchange interactions play a minor role and are only significant for rare earth ions lacking allowed absorption bands.^52^ Therefore, for Er^3+^–Cr^3+^ pairs in GdAlO_3_, multipolar interactions are expected to dominate the energy transfer process, provided there is suitable spectral overlap between their energy levels. The non-radiative energy transfer in Gd_0.99_Er_0.01_Al_0.995_Cr_0.05_O_3_ is taken place through the following cross-relaxations: (^2^H_11/2_ + ^4^A_2_(^4^F) → ^4^I_15/2_ + ^4^T_2_(^4^F)), (^4^S_3/2_ + ^4^A_2_(^4^F) → ^4^I_15/2_ + ^4^T_2_(^4^F)) transitions and *via* resonant phonon-assisted energy transfer (Fig. 19). This energy transfer process involving Er^3+^ ions explain the strong luminescence quenching of the emission bands at 542 nm, 521 nm and the appearance of an intense emission line at 726 nm when Cr^3+^ is incorporated. The decay of the emission at 697 nm under 377 nm excitation in Gd_0.99_Er_0.01_AlO_3_ is bi-exponential with lifetimes *τ′* = 2.61 ms. and *τ*′′ = 1.18 ms. Biexponential decay indicates that there are two different mechanisms that affect the decay dynamics and energy transfer may be one of these mechanisms. The decay curve shows two lifetimes: a longer time 2.61 ms (no transfer), a shorter time 1.18 (efficient transfer). This fact indicates that there is energy transfer from intrinsic defects to Er^3+^ ions in Gd_0.99_Er_0.01_AlO_3_. However, the decay curve at 697 nm under 377 nm excitation in Gd_0.99_Er_0.01_Al_0.995_Cr_0.05_O_3_ is described by a double-exponential equation *I*(*t*) = *I*_01_ exp(−*t*/*τ*_1_) + *I*_02_ exp (−*t*/*τ*_2_), where *I* is the luminescence intensity; *I*_01_ = 1844 and *I*_02_ = 1258 are constants; *t* represents time, *τ*_1_ = 1.69 ms and *τ*_2_ = 10.77 ms are decay times for the respective exponential components. The absence of the bands at 565 nm and 413 nm in the PLE spectrum of Gd_0_._99_Er_0_._01_AlO_3_ monitored at 697 nm indicates that the 697 nm emission line arise not only from electron transition between intrinsic defects centers, but also from the transition ^2^T_1_(^2^G)→^4^A_2_(^4^F) of Cr^3+^. This assignment is justified since the experimental value 697 nm (14 347 cm^−1^) is well reproduced by theoretical value of the transition in Table 2. Moreover, the highest-value lifetime *τ*_2_ = 10.77 ms is characteristic of the spin-forbidden transition. *τ*_1_ = 1.69 ms is fluorescence lifetime of 697 nm emission coming from the electron transition between two defects centers. The co-doping with chromium leads to a reduction of fluorescence lifetime of 697 nm emission from 2.61 ms to 1.69 ms, indicating that there is an increase in the transfer rate *K*_T_ constant. A dramatic decrease in the 697 nm emission line intensity and the appearance of an intense emission line at 726 nm support the energy transfer from oxygen-vacancies to Cr^3+^ ions *via* resonant phonon-assisted energy transfer from oxygen defect to Cr^3+^ : ^4^T_2_(^4^F) level and cross-relaxation processes (Fig. 19). The value of the constants *I*_01_ and *τ*_1_ in the PL decay at 697 nm under 377 nm excitation in Gd_0.99_Er_0.01_Al_0.995_Cr_0.05_O_3_ indicate that a very low part *I*_01_ exp(−*t*/*τ*_1_) of the emission at 697 nm is originating from electron transition between two intrinsic defects centers coupled to the B_3g_ (4) and B_1g_(7) vibrational modes. The highest part *I*_02_ exp (−*t*/*τ*_2_) of the emission at 697 nm inGd_0.99_Er_0.01_Al_0.995_Cr_0.05_O_3_ is originating from the transition ^2^T_1_ (^2^G) →^4^A_2_ (^4^F) of Cr^3+^. The energy transfer from Er^3+^, defects centers to Cr^3+^ and the high multiphonon relaxation from ^2^T_1_ (^2^G) to the ^2^E (^2^G) level, induces the intense line at 726 nm through the transition ^2^E (^2^G) →^4^A_2_ (^4^F) of Cr^3+^ (Fig. 19). Based on the experimental results and the theoretical optical considerations, the energy transfer process between Er^3+^, Cr^3+^ and oxygen-vacancies under *λ*_ex_ = 377 nm is presented in Fig. 19 and (Table 3).

**Table 3:** Fluorescence lifetime of Gd_0.99_Er_0.01_AlO_3_ and Gd_0.99_Er_0.01_Al_0.995_Cr_0.05_O_3_ samples, monitored at Er^3+^ emission wavelengths of *λ*_em_ = 542 nm and *λ*_em_ = 697 nm under *λ*_ex_ = 377 nm

Samples	Gd_0.99_Er_0.01_AlO_3_		Gd_0.99_Er_0.01_Al_0.995_Cr_0.05_O_3_	
The recorded wavelength	697 nm	542 nm	697 nm	542 nm
	Double-exponential	Mono-exponential	Double-exponential	Double-exponential
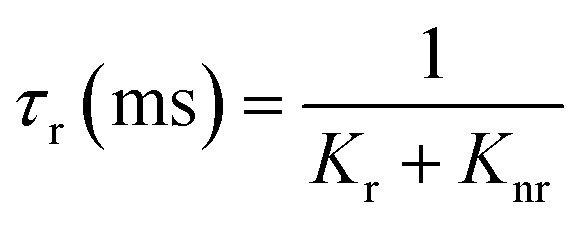	2.61–1.18	0.13	—	—
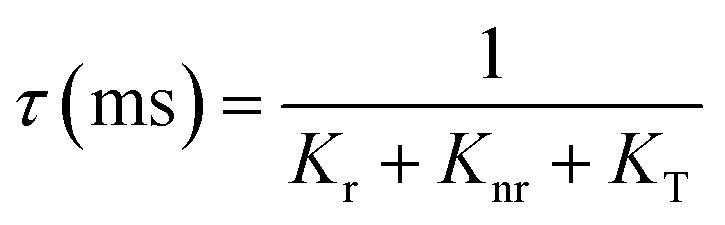	—	—	1.69–10.77	0.13–0.03

## 4. Conclusion

Gd_0.99_Er_0.01_AlO_3_ and Gd_0.99_Er_0.01_Al_0.995_Cr_0.05_O_3_ samples were successfully synthesized using the solid-state reaction method. XRD analysis confirmed that both samples crystallize in an orthorhombic structure with a *Pbnm* space group. Using the Derivation of absorption spectrum fitting (DASF) and the first derivative of reflectance d*R*/d*λ* methods, the optical band gaps were determined to be 5.93 eV and 5.90 eV, respectively. Red emission peaks at 680, 697, 705, 717, and 758 nm were observed in both samples and are ascribed transition involving intrinsic defects coupled with the B_3g_ (4) and B_1g_(7) vibrational modes. Co-doping with Cr^3+^ induced a significant decrease in Er^3+^ emission intensity, particularly the intensity of red lines at 680, 697, 705, 717 nm. The weak peak at 693 nm and the intense peak at 726 nm are assigned to the ^2^T_1_ (^2^G) → ^4^A_2_ (^4^F) and ^2^E_g_ (^2^G) → ^4^A_2g_ (^4^F) transitions of Cr^3+^. PL spectra and decay curves at 697 nm and 542 nm under 377 nm excitation confirmed efficient non-radiative energy transfer from Er^3+^ and intrinsic defects to Cr^3+^ ions. The energy transfer occurs *via* resonant phonon-assisted processes from the Er^3+ 2^H_11/2_ and ^4^S_3/2_ levels to the Cr^3+^ : ^4^T_2_ (^4^F) level, followed by cross-relaxations (^2^H_11/2_+^4^A_2_(^4^F) → ^4^I_15/2_+^4^T_2_(^4^F)),(^4^S_3/2_+^4^A_2_(^4^F) → ^4^I_15/2_+^4^T_2_(^4^F)). Decay curves at 697 nm indicate that the lower part of this emission originates from transition involving intrinsic defects coupled with vibrational modes, while the highest part of the emission at 697 nm in Gd_0.99_Er_0.01_Al_0.995_Cr_0.05_O_3_ is due to the Cr^3+ 2^T_1_ (^2^G) →^4^A_2_ (^4^F) transition. Finally, energy transfer from Er^3+^, defect to Cr^3+^and the high relaxation from ^2^T_1_ (^2^G) to the ^2^E (^2^G) level induce the intense line at 726 nm due to the transition ^2^E (^2^G) →^4^A_2_ (^4^F) of Cr^3+^.

## Author contributions

F. Mselmi: Conceptualization, methodology, writing – original draft, formal analysis. Abir Hadded: Writing – original draft, formal analysis, software. Hajer Souissi: Investigation, validation. Souha Kammoun: Investigation, validation, supervision. J. Pina: Software, investigation. B. F. O. Costa: Investigation, validation, supervision.

## Conflicts of interest

This research did not receive any specific grant from funding agencies in the public, commercial, or not-for-profit sectors.

## Data Availability

All the data for the manuscript is available in the manuscript.
